# Unveiling the fluorescence lifetime changes of a solvatochromic aurone probe dye upon wetting in an emissive polymer brush matrix by deconvolution of TCSPC data

**DOI:** 10.1140/epje/s10189-026-00597-5

**Published:** 2026-07-16

**Authors:** Jonah L. Decker, Sören Steup, Sergey I. Druzhinin, Heiko Ihmels, Holger Schönherr

**Affiliations:** 1 Physical Chemistry I, Department of Chemistry and Biology, and Research Center of Micro- and Nanochemistry and (Bio)Technology (Cμ), University of Siegen, Adolf-Reichwein-Str. 2, 57076 Siegen, Germany; 2 Organic Chemistry II, Department of Chemistry and Biology, and Research Center of Micro- and Nanochemistry and (Bio)Technology (Cμ), University of Siegen, Adolf-Reichwein-Str. 2, 57076 Siegen, Germany

## Abstract

The wetting processes of polymer brushes, embedded with a fluorescent probe, were investigated with time-correlated single photon counting. A suitable aurone dye was synthesized, which exhibits moderate fluorescence in aprotic solvents, poor fluorescence in protic solvents and practically no fluorescence in aqueous solution. Poly(diethylene glycol methyl ether methacrylate) (PDEGMA) brushes of ca. 200 nm thickness were obtained by polymerization from a Ti surface by surface-initiated atom transfer radical polymerization (SI-ATRP). The dye-loaded brushes show fluorescence of the dye comparable with the autofluorescence of the PDEGMA brushes and the subjacent α-bromoisobutyryl bromide (BiBB) functionalized polydopamine (PDA) layer. With a global analysis of fluorescence decays, the lifetimes of the complex multicomponent materials were deconvoluted. The dry polymer loaded with the dye possesses a five-exponential fluorescence decay with the characteristic decay times of ~ 3.0 ns for the dye, and ~ 6.0, 1.8 and 0.45 ns for PDEGMA brushes as well as < 0.05 ns for the PDA-BiBB layer. Because of the strong decrease of the fluorescence lifetime of the dye down to ~ 2.4 ns when submersed in water and a concomitant decrease of the lifetimes of the matrix to 4.5, 1.3 and 0.40 ns, both dye and matrix fluorescence are feasible reporter probes for the analysis of wetting processes of polymers with fluorescence lifetime imaging. The contrast of the intensity images of the aurone, which is low because of background fluorescence, increases by a factor of 3.1 in the time-resolved imaging at certain time delay after excitation. Hence, the incorporation of the aurone in the polymer brushes is a promising and versatile system for the investigation of (de)wetting dynamics employing fluorescence lifetime imaging and nanosecond time-resolved fluorescence imaging microscopy.

**Graphical abstract:**

**Figure:**
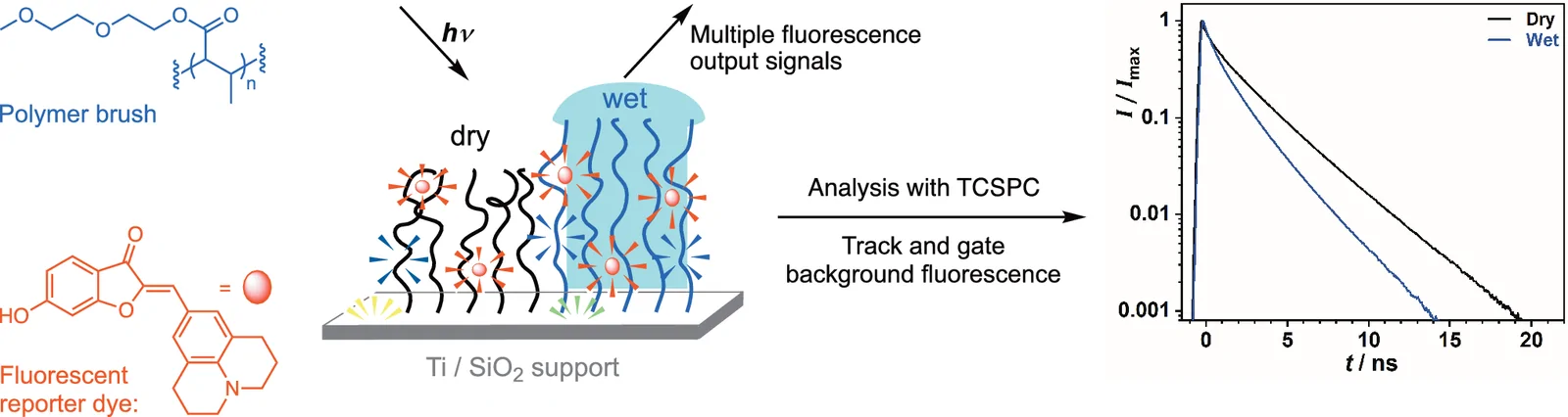
Incorporation of a fluorescent dye in polymer brushes allows monitoring of (de)wetting dynamics with time-resolved fluorescence imaging microscopy

**Supplementary Information:**

The online version contains supplementary material available at 10.1140/epje/s10189-026-00597-5.

## Introduction

The understanding and control of wetting and dewetting of liquids on surfaces is of paramount importance for a large variety of technologically relevant applications [1–3]. For dynamic wetting and dewetting processes on flexible, adaptive and switchable surfaces a complete description is currently far from being accessible. Such surfaces or thin layers may be affected by capillary interactions, which in turn has an impact on the static and dynamic behavior of the liquid [4, 5]. As shown before with various examples, the substrate dynamics are indeed coupled to the hydrodynamics in the wetting liquid and therefore provide additional time and length scales [1, 6].

In substrate supported (ultra)thin films as well as so-called brushes [7], densely grafted end-tethered polymer chains on a solid support, of water swellable or partially soluble polymers the experimental characterization of the wetting dynamics and the water diffusion into the films, which lead to swelling and thus altered (de)wetting dynamics, is complicated by the rapid processes on the relevant length scales as well as a lack of depth-sensitive techniques. The application of fluorescent tracer or probe molecules comprises in this context an interesting avenue to obtain more knowledge about these processes.

The analysis of molecular fluorescent probes partitioned in very low concentration in polymer films is a well-established, highly sensitive technique that provides insights into local environment, polymer dynamics, and structural integrity of these films. These fluorescent dyes function as probes that, upon excitation, emit light that can be analyzed to determine parameters like film thickness, glass transition temperature, and molecular diffusion rates [8–13]. Similarly, polymers that were end-labeled with a fluorophore have been employed to reveal differences associated to the local nanoscale surrounding of the probes in semi crystalline polymer films [14]. Likewise, the analysis of diffusion of small, low-molar-mass tracer molecules has been utilized to interrogate confinement effects in ultrathin (film thickness < 100 nm) substrate supported polymer films [15–17]. Examples are not limited to planar polymer films; also polymeric assemblies such as block copolymer vesicles have been analyzed by advanced time-resolved fluorescence methods. Handschuh-Wang et al. reported on the drying dynamics of polymer vesicles on the basis of fluorescence lifetime imaging microscopy (FLIM) measurements [18, 19].

Among the various fluorescent probes employed, solvatochromic probes are particularly suited to study the local polarity of their surroundings by shifts in fluorescence wavelengths [20, 21] or to respond to traces of water. For this purpose, specialized PET-type fluorescent monomers or carbon dots were embedded in polymer films (e.g., poly(methyl methacrylate)) to detect trace water through fluorescence quenching or color changes [22]. The water content in hydrogel, such as copolymers of poly(*N*-isopropylacylamide), showing thermoresponsive wetting properties, was found to be manifested with fluorescence lifetime (*τ*) of the reporter dye ATTO 655 at low water concentration during its drying [23] as well as at high water contents associated with thermally induced (de)swelling of hydrogel [24]. Here, the *τ* value anticorrelates with the humidity of the medium.

In general terms, the absorption and emission properties of organic compounds may change in response to their interaction with the medium, which constitutes a basic concept in the development of medium-sensitive probes [25, 26]. Based on the corresponding change in absorption or emission and the straightforward spectrometric analyses of the sample, these probes are employed in a wide variety of applications such as environmental sciences, biology, and pharmacology [27–29]. For instance, suitable dyes are frequently used for the photometric detection of solvent polarity [30]. But as compared with photometric analysis, fluorescence spectroscopy is a much more sensitive method [31]. Hence, tailor-made fluorescent probes have been developed for the fluorimetric analysis of, for example, polarity, polarizability, or hydrogen bond-accepting (HBA) or hydrogen bond-donating (HBD) properties, with high local and temporal control, even in complex media and polymers [25–28, 30, 31]. In particular, the identification and characterization of solvents, binding sites in host molecules, or organized and constrained media, such as polymers, zeolites, etc., with fluorescent probes is a straightforward and efficient method, because the optical properties of a suitable dye may be strongly influenced by either non-specific or specific interactions with its surrounding (microscopic) environment [25–28, 30–32]. The above-mentioned solvent properties may be assessed based on the different emission properties of solvatochromic probes, namely the distinct solvent dependent emission energy (*ν*), lifetime (*τ*), and quantum yield (*Φ*). Specifically, the fluorescence intensity or lifetime may increase or decrease in response to changing environment, thus leading to “turn-on” or “turn-off” probes, with the latter effect being more specific to the analyte or medium. In addition, fluorophores, whose emission energy shift of the fluorescence band changes, represent a beneficial type of probes, as this change is also readily monitored. The majority of such fluorescent probes consists of a conjugated donor–acceptor-substituted π system (push–pull), that enables an intramolecular charge transfer electronic transition giving different dipole moments of the ground and excited states, *μ*_g_ and *µ*_e_ [33–35]. In turn, the different stabilization of the solvatochromic molecules in the ground and excited state by unspecific (e.g. dipole–dipole) or specific (H-bonding) solute–solvent interactions, accompanied by solvent relaxation leads to different emission properties [33–35]. Usually, the resulting bathochromic shift (positive solvatochromism) or hypsochromic shift (negative solvatochromism) of the emission band correlates with the solvent parameters, such as dielectric constant and refractive index, Reichardt’s empirical polarity scales *E*_*T*_(30) or variations thereof, hydrogen-bond donating or accepting ability, acceptor number (AN) and donor number (DN), the Kamlet-Abboud-Taft parameters *α* and *β*, or Catalán’s solvent acidity and solvent basicity [36]. With these correlations at hand the emission properties of the solvatochromic dye are an efficient tool to assess and characterize the physical properties of its surrounding medium in a distance of typically approx. 5 nm around the fluorophore.

In the context of wetting of polymers, fluorescent probes offer the opportunity to obtain more important insight in the wetting processes, especially as fluorimetric analysis of polymers has developed into a highly useful tool [37]. Indeed, examples of dye-labeled polymers have been provided that unravel pertinent information related to dynamic wetting and monitoring of the concomitant changes induced in the polymer (swelling, chain stretching or reorientation) upon penetration of the molecules of a liquid. For example, Li, Butt et al. analyzed the time scale until water diffused through a 45 nm thin polystyrene/poly(acrylic acid) film, as indicated by the quenching the fluorescence of a surface-immobilized pH-responsive probe [38]. In another approach, Choi and Kwak integrated a solvatochromic fluorescent dye into hydrophobic methyl methacrylate or styrene based polymers to obtain fluorescent materials and monitored their wettability with alcohols based on the resulting red-shifted emission [39]. These experiments also allowed the authors to show the reversibility of this process. Similarly, a surface-immobilized fluorescent probe was employed to characterize the air–solid interface under surface nanobubbles [40]. Furthermore, the separation between fluorophore-labeled polymer chains and a metal supporting substrate have been determined by surface plasmon fluorescence spectroscopy, hence providing access to monitor chain relaxation in the form of stretching/collapse of the polymer or orientational changes [41]. A pair of dyes, whose local separation is determined fluorimetrically by Förster-resonance-energy transfer (FRET), has also been covalently attached to stimulus-responsive polymer brushes by Besford, Fery et al. [42] In this setup, the changes of polymer conformation with varying composition of liquid mixtures may be determined in real time by fluorescence spectroscopy because of the resulting alteration of the distance between the dyes, and thus the FRET-based emission. More recently, a fluorescent polymer with photochromic spiropyran units has been reported whose wettability may be switched by light. But although the polymer was analyzed with its intrinsic fluorescence properties, the wetting process has not been followed fluorimetrically [43].

A general concern with the analysis of fluorophore probe molecules in the above mentioned context is the potential “parasitic” autoemission of additional fluorescent components, e.g. “contaminants” in the probe or tracer dye formulations, such as highly emissive reaction by-products, stabilizers for monomers, catalyst residues or emitters of unknown origin, which hampers especially time-resolved measurements carried out at ultralow dye concentrations that are necessary to prevent altering of the polymer as a result of the loaded dye.

In the current contribution we highlight the feasibility of the analysis of the ingress of molecules of a wetting liquid into a tracer dye-equipped polymer brush, exploiting (i) a novel fluorophore designed specifically to report sensitively the local state of solvation/ hydration and (ii) a data analysis approach that eliminates effectively time-resolved parasitic emission/matrix autofluorescence. The approach appears to be a generally applicable one and can be expanded to fluorophores conjugated to the polymer chains comprising the brushes.

## Methods

### Synthesis of aurone derivative 3a

(*Z*)-6-Hydroxy-2-((2,3,6,7-tetrahydro-1*H*,5*H*-pyrido[3,2,1-*ij*]quinolin-9-yl)methylene)benzofuran-3(2*H*)-one (**3a**). To a solution of 6-hydroxy-3-coumaranone (300 mg, 2.00 mmol) and 9-julolidine carboxaldehyde (603 mg, 3.00 mmol) in EtOH (25 mL) was added concentrated HCl (3 mL), and the reaction mixture was stirred at reflux for 3 d. After cooling to room temperature, the reaction mixture was poured into water (50 mL). The solid components were filtered off and washed with diethyl ether (2 × 50 mL) and water (10 mL). The crude product was crystallized from MeOH, and the product was obtained as dark purple crystals (562 mg, 1.69 mmol, 84%); mp < 270 °C (dec.). ^1^H NMR (600 MHz, DMSO-*d*_6_): *δ* = 1.93–1.84 (m, 4H, 2′-H, 7′-H), 2.71 (t, ^3^*J* = 6.3 Hz, 4H, 3′-H, 6′-H), 3.25 (t, ^3^*J* = 5.7 Hz, 4H, 4′-H, 5′-H), 6.57 (s, 1H, 10-H), 6.68 (dd, ^3^*J* = 8.4 Hz, ^4^*J* = 2.0 Hz, 1H, 5-H), 6.76 (d, ^4^*J* = 2.0 Hz, 1H, 7-H), 7.34 (s, 2H, 1′-H, 8′-H), 7.55 (d, ^3^*J* = 8.4 Hz, 1H, 4-H), 10.96 (s, 1H, OH). – ^13^C NMR (150 MHz, DMSO-*d*_6_): *δ* = 21.0 (C3′, C6′), 27.2 (C2′, C7′), 49.3 (C4′, C5′), 98.4 (C7), 112.5 (C5), 113.2 (C10), 113.8 (C9), 117.9 (C9′), 120.7 (C10′, C12′), 125.3 (C4), 130.6 (C1′, C8′), 144.3 (C2), 144.5 (C11′), 165.4 (C8), 166.7 (C6), 180.4 (C3). – MS (ESI^−^): *m/z* (%) = 332 (100) [M^−^–H]. – El. Anal. for C_21_H_19_NO_3_, calcd. (%), C 75.66, H 5.74, N 4.20, found (%) C 75.69, H 5.82, N 4.22.

Synthesis of aurone derivative **3b** and **3c** are described in the Supporting Information (SI).

### Synthesis of PDEGMA brushes

The substrate preparation, polydopamine (PDA) deposition, α-bromoisobutyryl bromide (BiBB) attachment and polymerization of the poly(diethylene glycol methyl ether methacrylate) (PDEGMA) brushes was performed with slight modifications as described previously [44]. Details are provided in the SI.

### Dye loading of the brushes

To enable diffusion of aurone **3a** into the polymer brushes, the samples were immersed in a solution of the dye in absolute ethanol (500 nM). To prevent evaporation of the solvent, the samples were covered and stored by exclusion of light. After 18 h the samples were removed from the solution and dried in a nitrogen stream. To further dry the samples, they were placed in a desiccator for 30 min at 20 °C and reduced pressure (10 mbar).

### Compound characterization

*NMR spectroscopy*. ^1^H NMR spectra were recorded with a JOEL ECZ 500 (^1^H: 500 MHz and ^13^C: 125 MHz) and a Varian VNMR-S600 (^1^H: 600 MHz and ^13^C: 150 MHz) at *T* = 25 °C. The ^1^H NMR and ^13^C{^1^H} NMR spectra were referenced to the residual proton signal of the solvent [DMSO-*d*_6_: δ(^1^H) = 2.50 ppm, δ(^13^C) = 39.52 ppm or CDCl_3_: δ(^1^H) = 7.26 ppm, δ(^13^C) = 77.16 ppm]. Structural assignments were made with additional information from gCOSY, gHSQC, and gHMBC experiments. The NMR spectra were processed with the software MestreNova 12.

*Mass spectrometry*. The mass spectra were recorded with a Finnigan LCQ Deca (driving current: 6 kV, collision gas: argon, capillary temperature: 200 °C, support gas: nitrogen) and processed with the software Xcalibur.

*Elemental analysis*. Elemental analysis data were determined in-house with a HEKAtech EuroEA combustion analyzer.

*Melting point*. The melting points were measured with a melting point apparatus BÜCHI 545 (Büchi, Flawil, CH) and are uncorrected.

### Absorption and emission spectroscopy

The absorption spectra were recorded with an Agilent Cary 3500 Compact UV–Vis spectrophotometer with Hellma quartz glass cuvettes 110-QS (*d* = 10 mm). The emission spectra were recorded on a Varian Cary Eclipse fluorescence spectrometer with Hellma quartz glass cuvettes 115 F-QS (*d* = 10 mm). All measurements were recorded at a temperature of *T* = 20 °C as adjusted with a thermostat. Additional information on the experimental procedure and the determination of the fluorescence quantum yield are provided in the SI.

### Ellipsometry

To analyze the dry thickness of the polymer brushes, an alpha-SE ellipsometer (J.A. Woollam Co., Inc., Lincoln, USA) was used. Three different incidence angles: 65°, 70°, and 75° of the light with wavelengths between 380 and 900 nm were used. More details on data analysis are provided in the SI.

### Dynamic water contact angle measurements

The dynamic water contact angle measurements were performed at 20 °C with a OCA-15 contact angle microscope (Dataphysics, Fildersatadt, Germany) with a 500 µL syringe (Hamilton, Bonaduz, Swiss) and the SCA20 software (version 5.0.22). More details on how the water contact angles were acquired are provided in the SI.

### X-ray photoelectron spectroscopy (XPS)

The XPS spectroscopy measurements were performed with a photoelectron/ESCA spectrometer (SSX-100 S-probe, Surface science instruments, Mountain View, USA) with 200 W of Al Kα X-ray radiation. The survey spectra were recorded with a measurement range from 0 to 1200 eV, with a resolution of 1 eV and a spot size of 800 µm^2^. The high resolution spectra were recorded with a resolution of 0.1 eV. The data processing was done with the Casa XPS software (version 2.3.25 PR 1.0).

### Grazing angle Fourier transform infrared spectroscopy

The Fourier transform infrared spectroscopy (FTIR) measurements were conducted with a Vertex Neo R spectrometer (Bruker Optics, Ettlingen, Germany) together with a VeeMAX-II Variable Specular Reflectance Accessory (PIKE Technologies, Fitchburg, USA). More details on data acquisition and processing are provided in the SI.

### TCSPC measurements

The time-correlated single photon counting (TCSPC) fluorescence decays were measured with a PicoQuant confocal fluorescence microscope comprising an OLYMPUS IX-71 frame (Olympus, Hamburg, Germany), a Microtime 200 main optical unit (PicoQuant, Berlin, Germany), a FCU II fiber coupling unit (Olympus, Hamburg, Germany), and a PicoHarp 300 data acquisition module (PicoQuant, Berlin, Germany). The polymer brushes were grown on the glass cover slips (borosilicate glass, 20 mm × 20 mm with a thickness of 0.16 mm, Menzel-Gläser, Germany) coated with 5 nm titanium. The samples were fixed in a sample holder prior to the measurements. For analysis of the brushes in water, Milli-Q water (1 mL) was added onto the cover slip in the sample holder. The solution of aurone in EtOH was placed in the well plate, sealed by a glass cover slip and irradiated at 30 µm above the bottom of the well. Samples were excited by pulsed laser LDH-D-C-485 (PicoQuant, Berlin, Germany) with a repetition rate of 20 MHz at 485 nm. The resolution was set to 16 and 4 ps per channel. A horizontal xy-scans (80 × 80 µm^2^) kept the irradiation dose low to reduce sample photobleaching during TCSPC data acquisition. Fluorescence was excited and collected through an air objective with 40× magnification (Olympus Plan N 40×/0.65, Hamburg, Germany). The fluorescence was detected with a single-photon avalanche diode (PDICTC, Micro Photon Devices, Bolzano, Italy). Images were recorded with the P-733.2xx objective microscope piezo scanning stage and E-710 digital piezo controller scanner (Physik Instrumente GmbH, Germany) with a pixel resolution up to 512 × 512. The parameters for the fluorescence decay [45, 46] of polymer brushes and aurone dye were obtained by employing a nonlinear gradient least-squares method, with the global sum of the squares of the weighted residuals $S = \mathop \sum \limits_{i} w_{i} \left( {y_{i} - y_{i}^{e} } \right)^{2}$ as the target function. In the expression for *S*, *w* is the weighting factor of the response function, *y* was calculated as sum of a convolutions of the instrument response function *p* measured as the excitation light scattering at the air/glass interface with a sum of exponentials (amplitude $a_{j}$ and decay time $\tau_{j}$) and the background *b* (Eq. 1).

1 $$ y\left( t \right) = p\left( t \right)*\left( {\mathop \sum \limits_{j = 1}^{l} a_{j} e^{{ - \frac{t}{{\tau_{j} }}}} } \right) + b $$

The indexes *i* and *j* count the experimental points for each of typically 60 response functions, usually ~ 3000, and components for each decay function, j = 1, 2, … 5, respectively. The superscript ‘*e*’ indicates that the quantity is an experimental value. For the fluorescence decay the factor *w* = *y*^−1^.

## Results and discussion

**Fig. 1:**
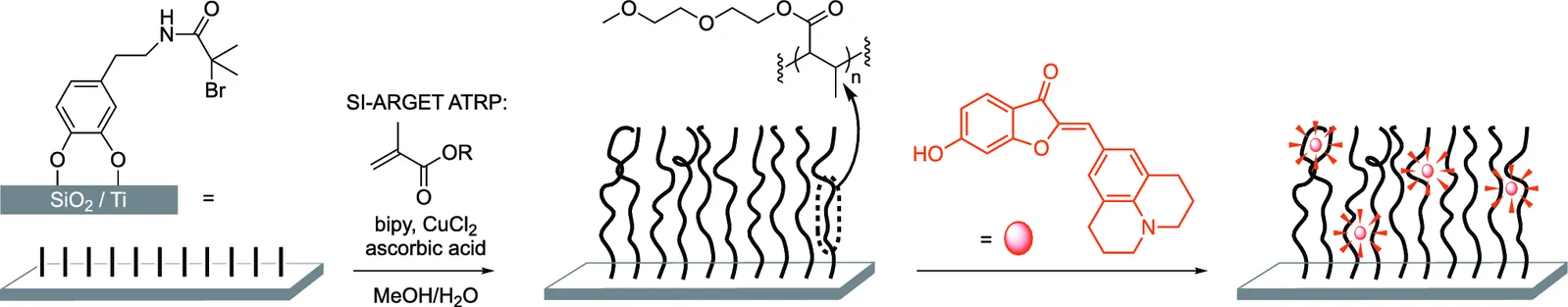
Schematic illustration of the sample fabrication, first step is the polymerization to poly(diethylene glycol methyl ether methacrylate) PDEGMA brushes from polydopamine, α-bromoisobutyryl bromide (BiBB) as initiator, second step is the loading of the brushes with the tracer dye

For the analysis of the local solvation state inside the brushes (compare Fig. 1) a solvatochromic dye for polymer functionalization was synthesized and fully characterized. The dye was subsequently loaded by diffusion into PDEGMA brushes on Ti, and static and dynamic fluorescence analyses were performed to confirm the suitability of the system to detect changes in local solvation.

### Synthesis of a solvatochromic dye for polymer functionalization

**Fig. 2:**
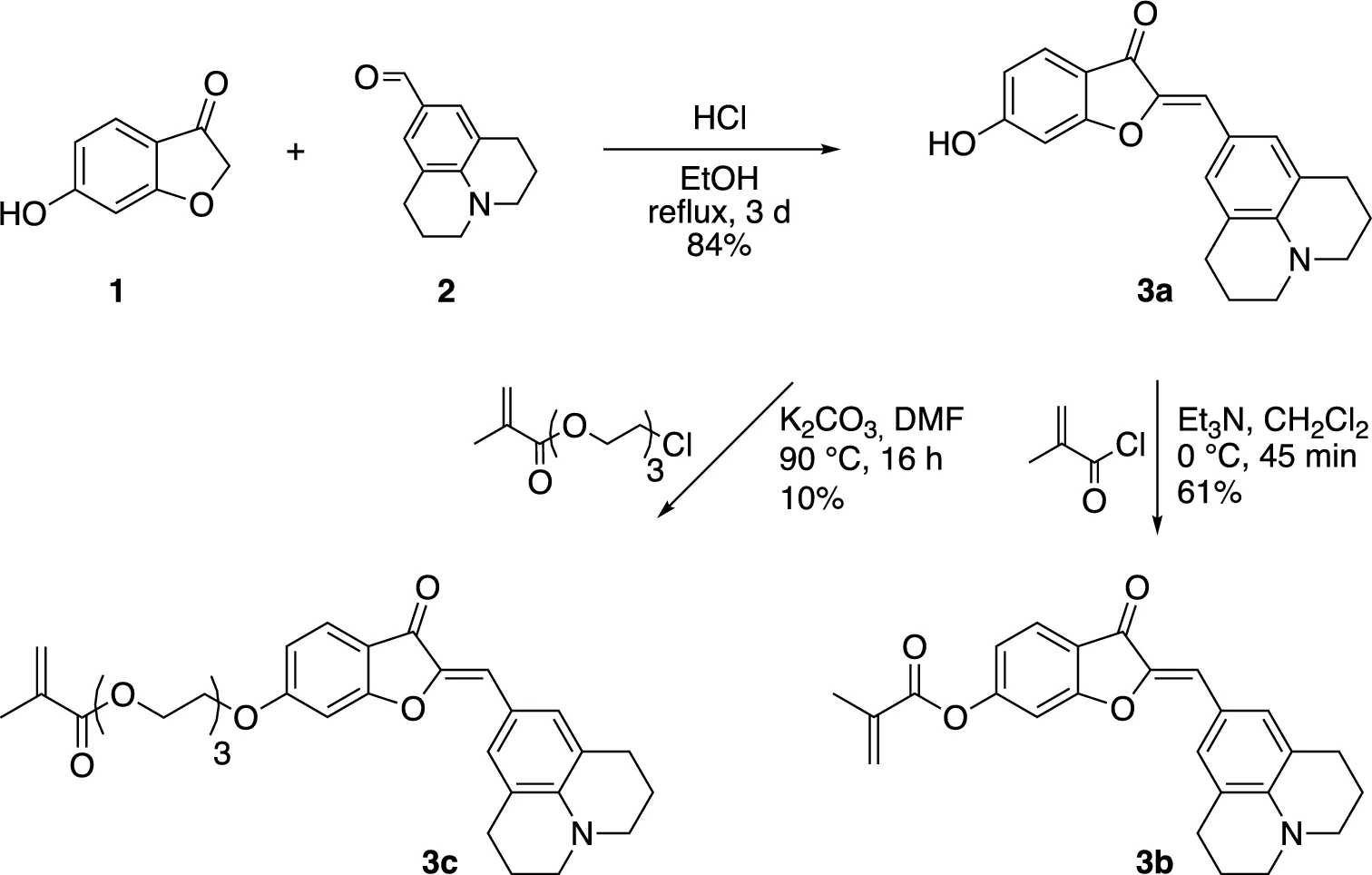
Synthesis of aurone derivatives **3a**–**c**

After screening of a series of solvatochromic dyes, donor-substituted aurones were chosen as model fluorophores. And to avoid interference of undesired effects on the emission properties by molecular relaxation, in particular quenching, the established julolidine was used as donor unit [47]. For this purpose, the aurone was synthesized in an acid-catalyzed condensation reaction [48] of 6-hydroxycoumaranon (**1**) with 9-julolidinylcarboxaldehyde (**2**) in 84% yield (Fig. 2). To examine whether this dye may also be used as part of monomer units for subsequent polymerizations in future applications, it was exemplarily attached to a methacrylate fragment. For direct attachment, the aurone **3a** was synthesized by esterification under basic conditions [49]. Unfortunately, at room temperature and longer reaction time (> 1 h) significant decomposition occurred. However, when the reaction temperature was kept at 0 °C and the reaction time was reduced to 45 min, the product **3b** was obtained in 61% yield (Fig. 2). In another approach, the methacrylate fragment was attached through a suitable linker to accomplish a reasonable separation between fluorophore and polymer-forming unit. For that purpose, the known 2-(2-(2-chloroethoxy)ethoxy)ethyl methacrylate [50] and the aurone **3a** were submitted to a Williamson ether synthesis in DMF with K_2_CO_3_ as base to give the “PEGylated” aurone-acrylate conjugate **3c** in 10% yield (Fig. 2). All novel compounds were identified and fully characterized by NMR spectroscopy (^1^H, ^13^C, COSY, HSQC, HMBC), melting point, and elemental analysis (cf. SI, Fig. S1–S6).

### Steady-state absorption and emission properties of aurone 3a

**Fig. 3:**
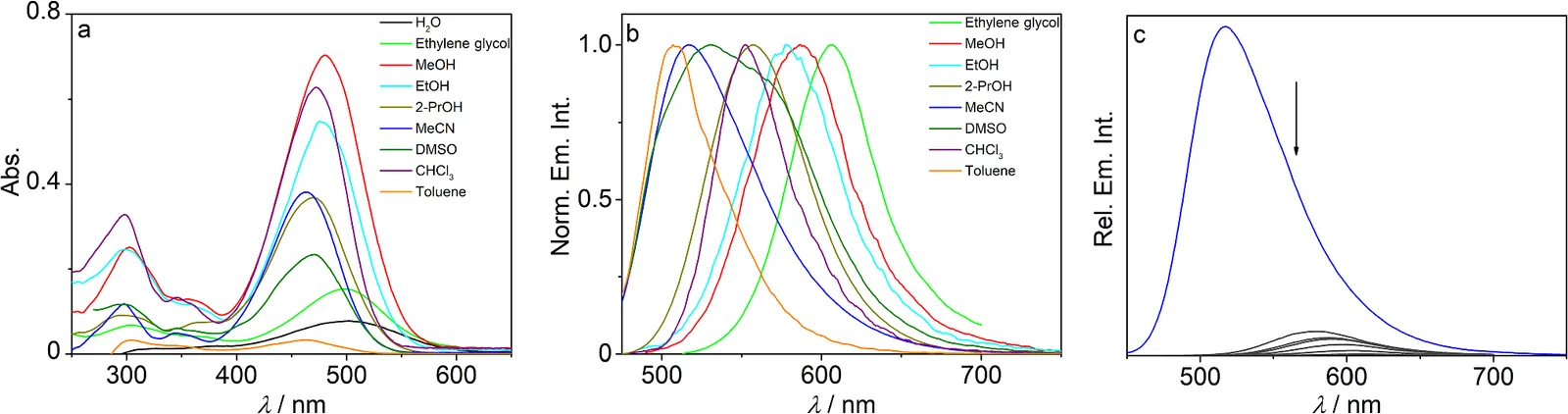
Absorption (**a**) and emission (**b**, **c**) spectra of **3a** (concentration 40 µM, except water) in water, ethylene glycol, methanol, ethanol, 2-propanol, acetonitrile, dimethylsulfoxide, chloroform, and toluene; solvents in order of decreasing solvent polarity. (**c**) fluorescence spectra of **3a** (*c* = 40 µM) in aqueous acetonitrile solutions with varying content of water (0–100% v/v); arrow indicates the development of fluorescence bands with increasing water content in the order 0% (blue), 5%, 15%, 20%, 40%, 60%, 80% (all dark gray), 100% (black)

The absorption and emission properties of aurone derivative **3a** were investigated in different solvents (Fig. 3). The maxima of the long-wavelength absorption band change only slightly in different solvents, namely from 480 nm in MeOH to 463 nm in toluene (Fig. 3a). As a general trend, the extinction coefficient of the dye decreases with lower polarity of the solvent. As the only outlier, the absorption was red shifted in water with an absorption maximum at 503 nm with very low extinction coefficient, presumably indicating aggregate formation because of the limited solubility of the dye in water. At the same time, the dependence of the fluorescence properties on the solvent are more pronounced. Firstly, as observed with resembling donor-substituted aurone derivatives [51], the hydroxy functionality in the 6-position causes a red shift of the fluorescence maxima in all solvents as compared with the fluorescence bands of aurones without a donor functionality at this position. In particular, the fluorescence maxima range from 587 nm in polar, protic solvents like methanol (MeOH) to 507 nm in nonpolar solvents such as toluene (Fig. 3b). Such a fluorosolvatochromic behavior is known for aurone derivatives and explained by a charge-transfer character of the excited state and resulting different stabilization of the excited state by solvents of varying polarity or hydrogen bond-donating or -accepting properties [52, 53]. Thus, attempts to correlate the fluorescence maxima of the aurone **3a**, given in wavenumbers, with common solvent parameters [27, 28, 30, 36] only resulted in a reasonable correlation with the acceptor number AN (*r*^2^ = 0.92) or the Kamlet-Abboud-Taft parameter α (*r*^2^ = 0.85) of the employed solvents (cf. SI, Fig. S11a, b). The acceptor number characterizes the ability of a solvent to stabilize a negative charge, whereas the Kamlet-Abboud-Taft parameter α relates to the hydrogen bond-donating properties of the solvent. This correlation of the fluorescence energy with the AN and α values of the solvent indicates the formation of a pronounced negative polarization in the excited state upon charge transfer, likely at the electron-accepting carbonyl group, and its concomitant stabilization by the solvent. However, this rather moderate degree of correlation, along with even much lower ones with other solvent parameters—even with the polarity scale *E*_T_(30) (*r*^2^ = 0.54) (cf. SI, Fig. S11c)—indicate a more complex solvent effect. Most likely, additional specific and non-specific solvent properties, such as polarity, polarizability, or the hydrogen bond accepting properties contribute to a minor extent and with different trends to the stabilization or destabilization of the ground and/or excited state of **3a** as well. And this interplay of different solvent effects cannot be dissected with the available data set. The fluorescence quantum yields of **3a** are very low in chloroform (CHCl_3_), acetonitrile (MeCN) or MeOH (< 0.01) and slightly higher in dimethylsulfoxide (DMSO) (0.20) and 2-propanol (0.33) (Table 1). Apparently, structural relaxation does not contribute significantly to the fluorescence quenching, because even in the most viscous solvent ethylene glycol the fluorescence quantum yield is low (0.08). In water, the fluorescence intensity of **3a** is too small to be detected as meaningful band, suggesting a strong quenching by the interaction of the excited molecule with the surrounding solvent shell [31]. Considering the low solubility of the dye in aqueous solution, however, the fluorescence quenching may also be caused by aggregation [31]. To further assess the effect of water on the fluorescence properties of **3a** in organic solvents, fluorescence spectra were determined in water-acetonitrile mixtures with different water content (cf. SI). Hence, with increasing water fraction the fluorescence maximum shifted from 515 nm to ca. 600 nm, and the intensity decreased strongly. Specifically, already at 5% water content the emission was quenched by 93%.

**Table 1:** Absorption and fluorescence of **3a** in different solvents

Solvent^a^	*λ*_abs_^b^/nm	*ϵ*^c^/mol L^−1^ cm^−1^	*λ*_fl_^d^/nm	*Φ*^e^	Stokes shift/cm^–1^
H_2_O	503	1940	n.d.^f^	n.d.^f^	n.d
Ethylene glycol	498	3820	606	0.08	3640
MeOH	480	17570	587	< 0.01	3870
EtOH	476	13690	578	0.06	3800
2-PrOH	470	9198	558	0.33	3440
MeCN	463	9530	515	< 0.01	2420
DMSO	470	2890	530	0.20	3000
CHCl_3_	473	15710	553	< 0.01	3110
Toluene	464	830	507	0.17	1920

^a^Solvents arranged in order of decreasing solvent polarity, according to solvent parameter *E*_*T*_*(30)*

^b^Absorption maximum at *c* = 40 μM (except water)

^c^Molar extinction coefficient

^d^Fluorescence maximum at *c* = 40 μM (except water), *λ*_*ex*_ corresponds to the absorption maximum

^e^Fluorescence quantum yield, relative to coumarin 153 (*Φ* = 0.38 in EtOH [54]), *λ*_*ex*_ = 421 nm, estimated error: ± 10% of the given values

^f^Not determined because of insufficient solubility

### Synthesis and characterization of PDEGMA brushes

**Fig. 4:**
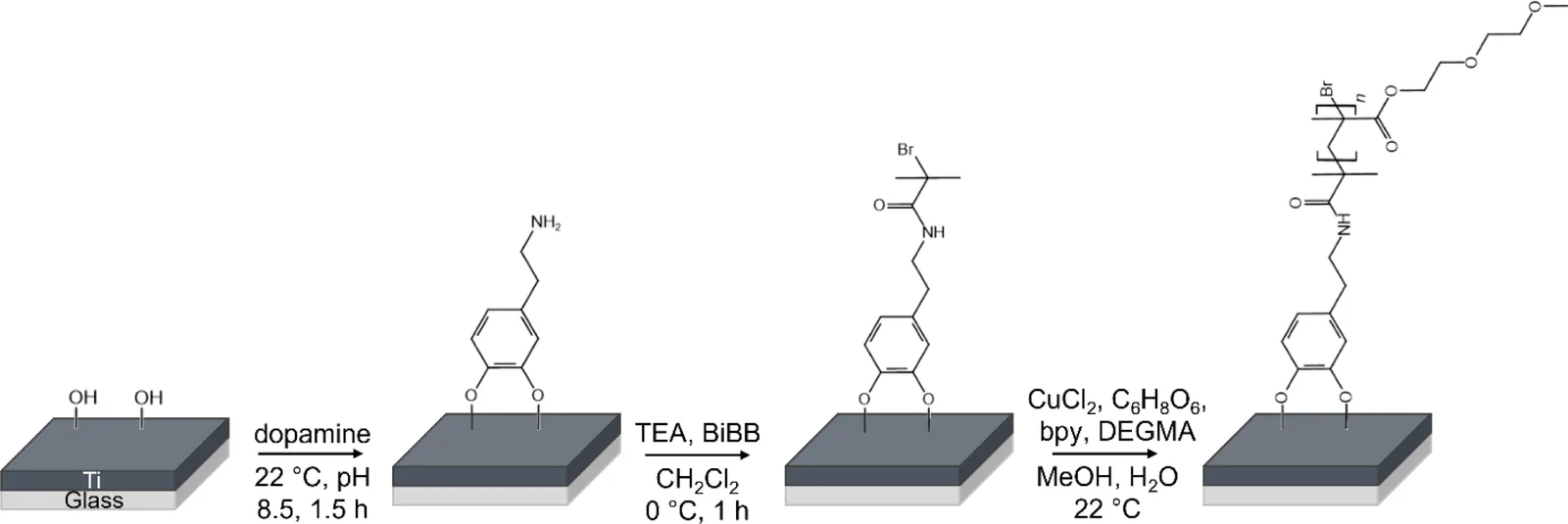
Schematic illustration of the PDEGMA brush synthesis on titanium substrates: Step 1: deposition of polydopamine; Step 2: attachment of BiBB as ATRP initiator; Step 3: DEGMA polymerization by SI-ARGET ATRP

PDEGMA brushes were synthesized by surface initiated activator regeneration by electron transfer atom transfer radical polymerization (SI-ARGET ATRP, Fig. 4) [44]. As anchoring layer, polydopamine (PDA) polymerized onto titanium substrates was used [44]. The polymerization of dopamine was performed in an aqueous solution for 1.5 h yielding into a 3.2 ± 0.2 nm thick PDA layer determined by ellipsometry. α-Bromoisobutyryl bromide (BiBB) was subsequently covalently coupled to the amine functionalities of polydopamine as ATRP initiator (Fig. 4). This resulted in an increase in ellipsometric thickness of 0.6 ± 0.1 nm.

Di(ethylene glycol) methyl ether methacrylate (DEGMA) polymerization by SI-ARGET ATRP was performed in a water/methanol mixture for 30 min to obtain brushes of 184 ± 9 nm dry ellipsometric thickness. The apparent dynamic water contact angle of each layer was determined by the sessile drop method, with which water was pumped into the droplet and sucked out of the droplet to determine the advancing and receding contact angles, respectively. While water is completely wetting the surface on a freshly cleaned titanium surface (cf. SI, Fig. S7), and no accurate value of the contact angle could be measured (contact angle < 5°), the advancing water contact angle on the polydopamine coated surface was 45.5° ± 4.6°, and the receding water contact angle was 5.4° ± 1.2°. By functionalizing the polydopamine with BiBB, the advancing (75.8° ± 2.2°) as well as the receding contact angle (25.4° ± 3.6°) increased (Table 2). The polymerization of PDEGMA brushes onto the initiation layer caused an increase of the contact anglehysteresis, as the advancing contact angle was increased and the receding contact angle was reduced (Table 2). The increase in the contact angle hysteresis is caused by conformational changes of the brushes, especially the ethylene glycol side chain adapting to the wetting. Additionally, increasing the layer thicknes from 3.8 to 184 nm also changes the surface roughness, which, in turn, has a direct effect on the contact angle hysteresis.

**Table 2:** Dynamic water contact angles of the polydopamine layer, polydopamine layer functionalized with BiBB, and the PDEGMA brushes

Contact angle^a^/°	PDA	PDA-BiBB	PDEGMA
Advancing	45.5° ± 4.6°	75.8° ± 2.2°	97.4° ± 1.0°
Receding	5.4° ± 1.2°	25.4° ± 3.6°	12.5° ± 0.8°

^a^Water contact angle determined by sessile drop method. Advancing and receding contact angles were determined by pumping in and sucking out water from the droplet

**Fig. 5:**
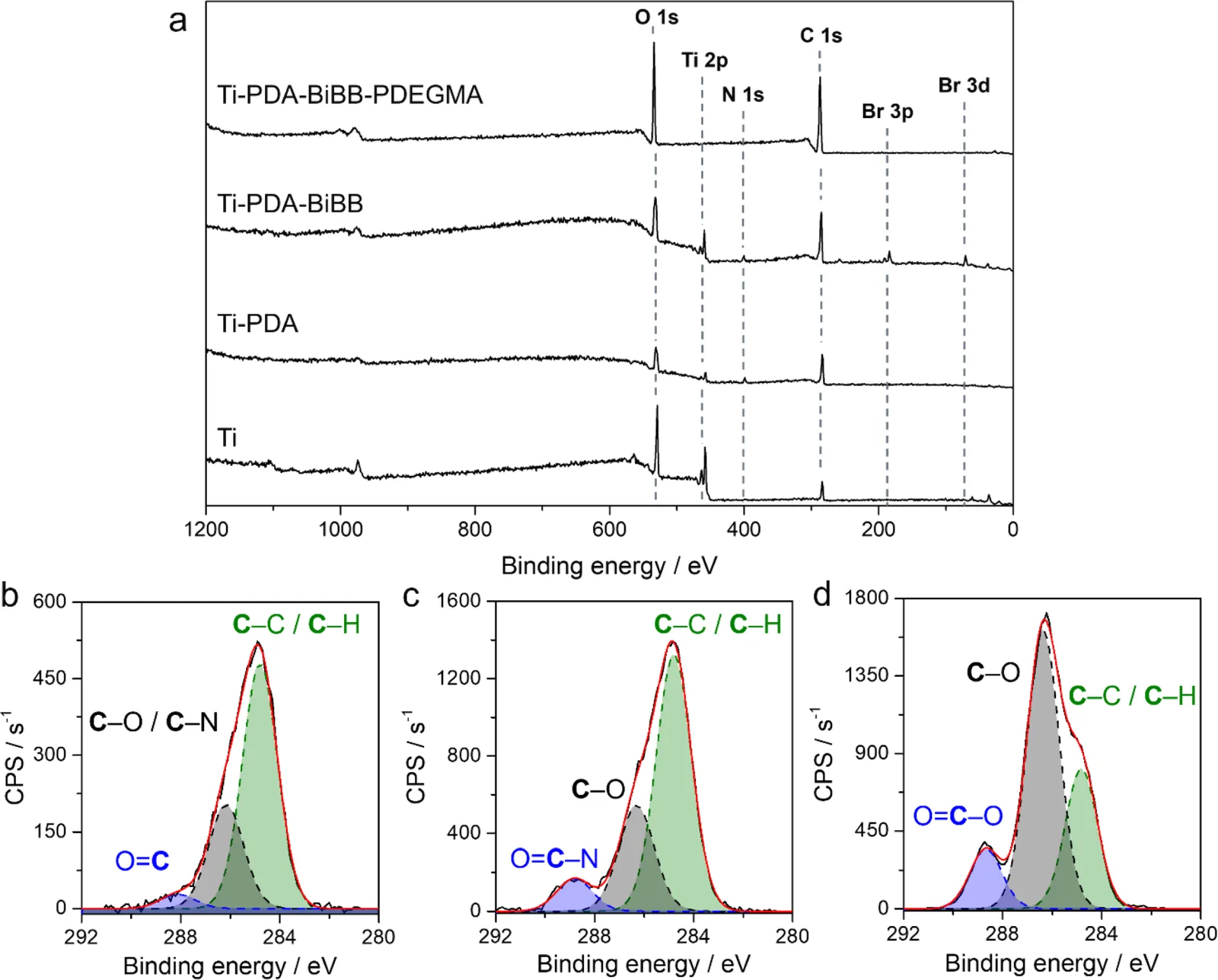
X-ray photoelectron spectra; (**a**) survey scans of bare Ti, Ti modified with polydopamine (PDA), Ti modified with PDA functionalized with BiBB and PDEGMA polymerized from PDA-BiBB; carbon 1*s* high resolution spectrum of (**b**) polydopamine; (**c**) PDA functionalized with BiBB; (**d**) PDEGMA polymerized from PDA-BiBB

The elemental composition of each layer was characterized by X-ray photoelectron spectroscopy (XPS). The XPS survey scan of the titanium sample showed the signals of Ti 2*p* and O 1*s* from the oxidized titanium surface. Additionally, a C 1*s* signal was detected, which was caused by contaminations adsorbed to the surface during sample preparation or transfer. The XPS survey scan of the titanium sample coated with PDA showed the expected signals of C 1*s*, O 1*s*, N 1*s* and Ti 2*p* at 285 eV, 532 eV, 400 eV and 458 eV, respectively (Fig. 5a). When the N/C ratio of the PDA layer was corrected for the carbon already occurring in the XPS survey scan of a bare cleaned titanium sample, it was with ~ 0.10 in agreement with previously reported values and close to the theoretical value (0.125) (Table 3) [44, 55]. When the polydopamine was functionalized with BiBB the signals for bromine Br 3*p* and Br 3*d* were observed in the XPS survey spectrum at 183 eV and 70 eV. Additionally in the C 1*s* high resolution spectrum the signal for carbon in the amide functionality of PDA-BiBB (288.8 eV) was deconvoluted (Fig. 5c). In the XPS survey spectrum of the PDEGMA brushes only the C 1*s* and O 1*s* signals were detected, as the thickness of the brushes (~ 180 nm) by far exceeded the escape depth of the generated photoelectrons. The O/C atomic ratio (0.45) fits to the theoretical ratio (0.44) for PDEGMA.

**Table 3:** Atomic concentrations (atom-%) of titanium, carbon, oxygen, nitrogen and bromine obtained by XPS on bare Ti, Ti modified with polydopamine (PDA), Ti modified with PDA functionalized with BiBB and PDEGMA polymerized from PDA-BiBB

Signal	Ti	Ti-PDA	Ti-PDA-BiBB	PDEGMA
Ti 2*p*	15.7	2.5	4.3	–
C 1*s*	34.0	64.9	60.1	69.2
O 1*s*	50.3	26.5	28.3	30.8
N 1*s*	–	6.1	3.7	–
Br 2*p*	–	–	3.6	–

The C 1*s* high resolution scan of polydopamine was deconvoluted into three peaks: **C**–C at 284.8 eV, **C**–O/**C**–N at 286.2 eV and **C**=O at 288.1 eV (Fig. 5b) [56]. The carbonyl carbon peak was caused by a dopamine quinone that was formed as an intermediate during its polymerization [3]. The functionalization of PDA with BiBB introduced a carbon peak of the amide functionality (O=**C**–N) at 288.8 eV (Fig. 5c). The carbon high resolution scan of PDEGMA (Fig. 5d) was deconvoluted into three peaks: **C**–C at 284.8 eV, **C**–O at 286.4 eV and O=**C**–O at 288.7 eV with a ratio of 1:4.6:2.4 being close to the theoretical ratio of 1:5:3. Additionally the high resolution scans of O 1*s* and N 1*s* were analyzed (cf. SI, Fig. S8). All of the spectra were deconvoluted into the expected peaks in agreement with literature [44, 55, 56].

**Fig. 6:**
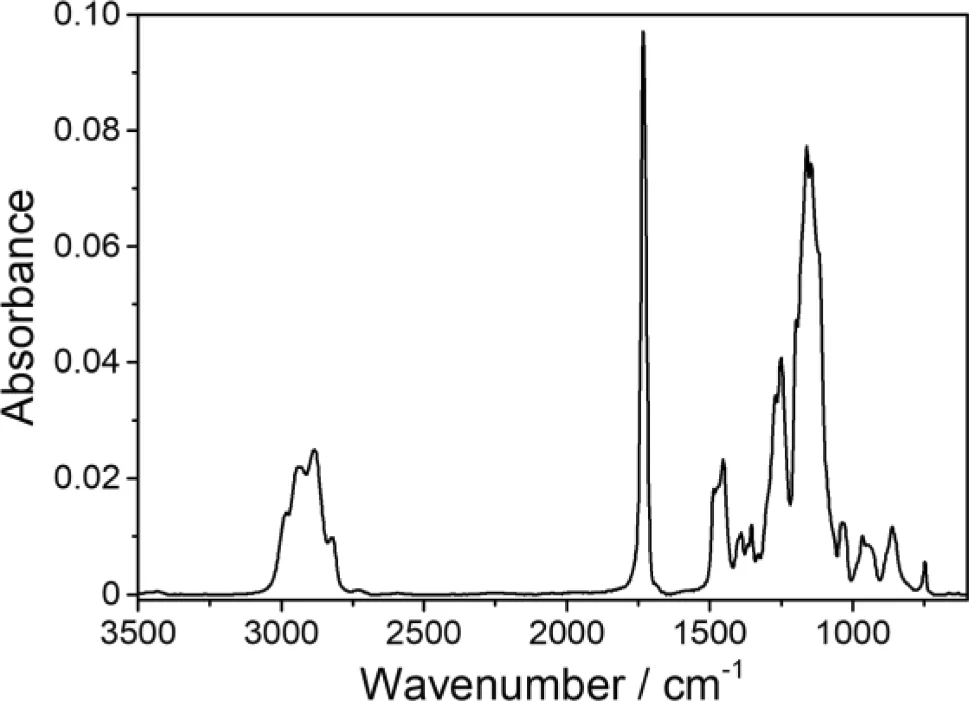
Grazing angle Fourier transform infrared (FTIR) spectrum of ~ 180 nm thick PDEGMA brushes polymerized from PDA-BiBB

Finally the PDEGMA brushes were analyzed by grazing angle FTIR spectroscopy (Fig. 6). Between 3000 and 2800 cm^−1^ the symmetric and asymmetric C–H stretching vibrations of CH_3_ and CH_2_ groups were observed. At 1730 cm^−1^ is the C=O stretching vibration of the carbonyl groups. The C–O–C stretching vibration was observable at 1145 cm^−1^; all observed vibrations were in agreement with previously published data [57].

### Fluorescence decay of dye-loaded PDEGMA brushes

The dye-loaded polymer brushes consist of a stratified layer of the PDEGMA brush grown from the underlying BiBB and polydopamine layers (Fig. 4). The dye was assumed to distribute within the entire polymer film and to potentially partition into the sublayers. Each of these sublayers materials may possess own fluorescence and/or autofluorescence, characterized by the corresponding sets of fluorescence lifetimes.

**Fig. 7:**
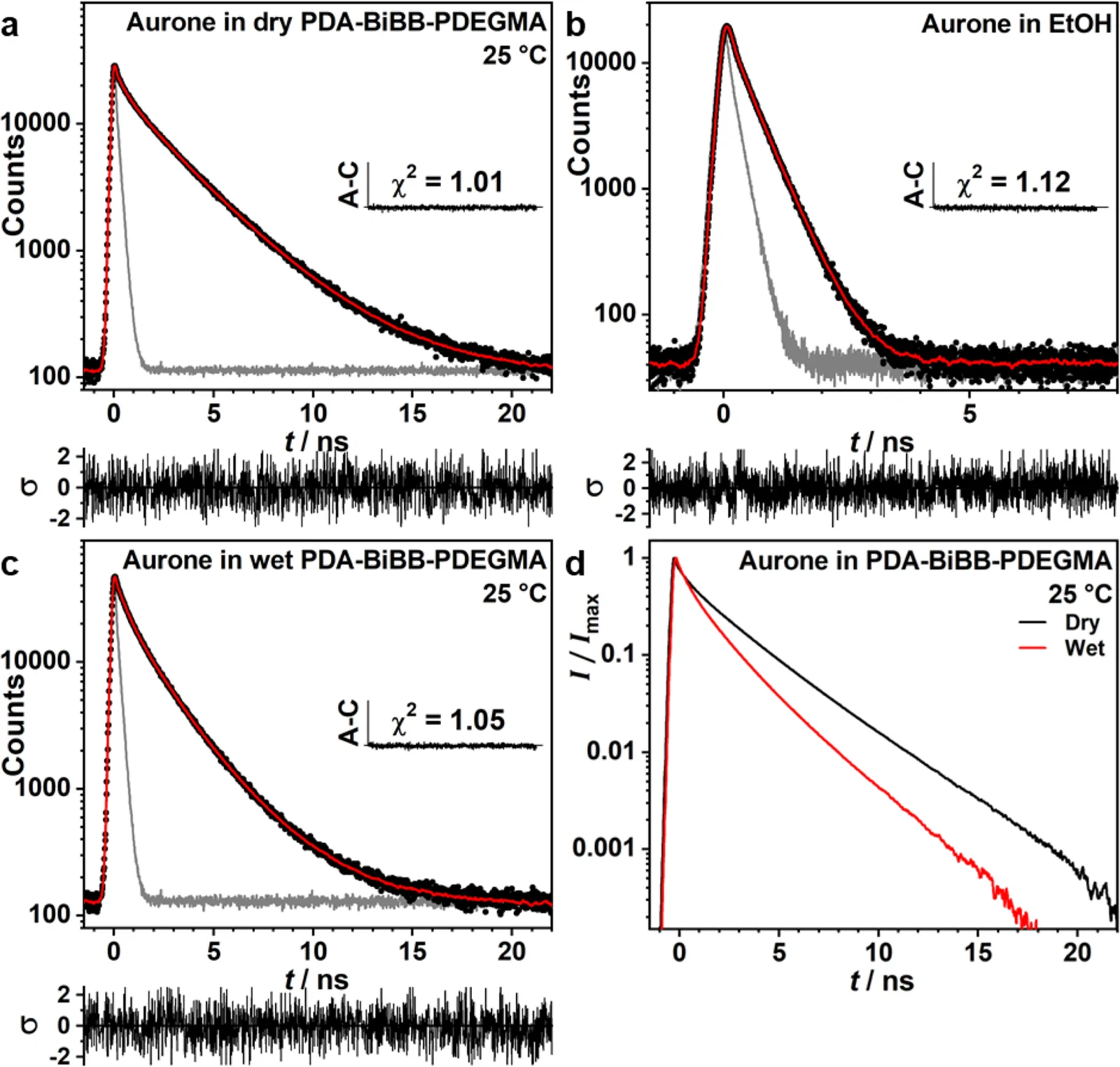
(**a**) TCSPC fluorescence decay (TCSPC decay) of PDA-BiBB-PDEGMA brushes (batch 2) loaded with aurone **3a** analyzed in air (dry) at 25 °C. (**b**) TCSPC decay of 500 nM aurone **3a** dissolved in EtOH at 22 °C. (**c**) TCSPC decay of PDA-BiBB-PDEGMA brushes (batch 2) loaded with aurone **3a** submersed in water (wet) at 25 °C. TCSPC panel: fluorescence decay, black dots, instrument response function (IRF), gray solid line, and fit, red solid line, (top panel) with the corresponding residuals (bottom panel) and autocorrelation function plot (inset in the top panel) are shown. In the panel, the χ^2^ value is provided. (**d**) An overlay of the normalized fluorescence decays depicted in panels (**a**) and (**c**). The excitation wavelength is 485 nm

**Fig. 8:**
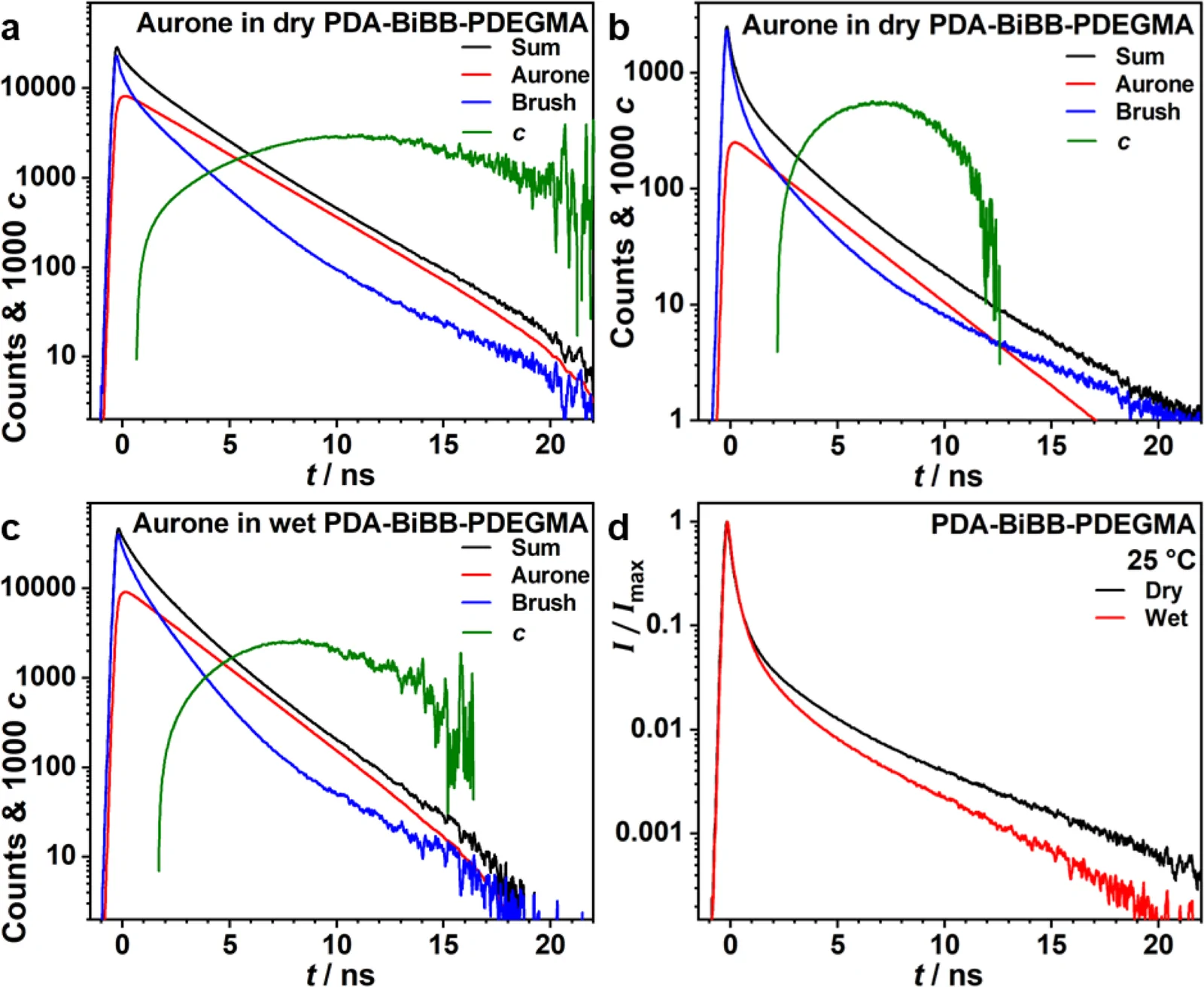
(**a**, **b**) Fluorescence decays of two different batches of PDA-BiBB-PDEGMA brushes loaded with aurone **3a**, batch 2 (**a**) and batch 1 (**b**), both analyzed in air (dry) at 25 °C and 22 °C, respectively. (**c**) Fluorescence decay of PDA-BiBB-PDEGMA brushes, batch 2, loaded with aurone **3a,** submersed in water at 25 °C. The calculated components of the fluorescence kinetics correspond to aurone **3a** (*τ*_*d*_), polymer brushes (*τ*_*p*1_, *τ*_*p*2_, *τ*_*p*3_ and *τ*_*p*4_) as well as the sum of them. The aurone/brush image contrast function *c* was calculated with Eq. (5). (**d**) Overlay of the normalized fluorescence decays of PDA-BiBB-PDEGMA brushes batch 2 in air (dry) and submersed in water (wet) at 25 °C. The excitation wavelength is 485 nm

The corresponding fluorescence decays of PDA (thickness 3.2 nm), PDA-BiBB (BiBB thickness 0.6 nm), PDA-BiBB-PDEGMA (PDEGMA thickness 184 nm) films, in pristine form as well as loaded with the reporter dye aurone derivative **3a** (500 nM in EtOH) were investigated employing a time-resolved confocal fluorescence microscope. In this set-up, the axial spatial resolution of ~ 1.4 µm is substantially larger than the overall polymer film thickness of about ~ 200 nm, independent whether the film is wetted with water or not. Therefore, all components of the polymer layer were excited practically simultaneously and evenly within the cross-section of the film. The decays measured for the various samples (see above) under different conditions and aurone-doped brushes for different batches of samples are summarized in Figs. 7 and 8, and will be discussed subsequently.

The time-resolved (auto)fluorescence of the film components were collected together merging into rather complex fluorescence decay *I*(*t*) of the entire film according to Eq. (2).

2 $$ I\left( t \right) = y\left( t \right) - b $$

A convolution (compare Eq. 1) was employed to fit the TCSPC fluorescence decays. While five exponential terms were required to fit the most complex fluorescence decay of PDA-BiBB-PDEGMA brushes loaded with aurone **3a** (Fig. 7a and Table 4), a four-exponential-terms function was sufficient to approximate the fluorescence decay of the PDA-BiBB-PDEGMA brushes in the absence of tracer dye. A somewhat unexpected observation was the strong short-lived emission of supported PDA and BiBB layers. Its decay time was shorter than the time resolution of the microscope (~ 50 ps) and, therefore, the fluorescence kinetics of PDA and BiBB could not be differentiated in the present experiment.

**Table 4:** Fluorescence decay of PDA-BiBB-PDEGMA^a^ polymer brushes loaded with aurone **3a** at 22 °C (unless stated otherwise)

Material	*τ*_p1_^b^/ns (*a*_1_^c^)	*A*_1_^d^/%	*τ*_p2_^b^*/*ns (*a*_2_^c^)	*A*_2_^d^*/*%	*τ*_p3_^b^*/*ns (*a*_3_^c^)	*A*_3_^d^*/*%	*τ*_p4_^b^*/*ns	*A*_4_^d^*/*%	*τ*_d_^e^*/*ns (*a*_d_^c^)	*A*_d_^f^/%
**3a** in PDA-BiBB-PDEGMA^g^	5.97 (0.047)	8.0	1. 76 (0.53)	26	0.43 (1)	12	< 0.05	20	3.02 (0.38)	49
PDA-BiBB-PDEGMA^g^	5.97 (0.040)	12	1.76 (0.16)	14	0.43 (1)	22	< 0.05	52	–	–
PDA-BiBB	–	–	–	–	–	–	< 0.05	100	–	–
PDA	–	–	–	–	–	–	< 0.05	100	–	–
**3a** in PDA-BiBB-PDEGMA^h^	6.08 (0.046)	2.9	1.90 (1.6)	32	0.47 (1)	4.9	< 0.05	8.4	3.12 (1.6)	109
PDA-BiBB-PDEGMA^h^	6.08 (0.091)	19	1.90 (0.30)	19	0.47 (1)	16	< 0.05	46	–	–
**3a** in PDA-BiBB-PDEGMA^i^	4.46 (0.046)	2.9	1.27 (1.5)	38	0.39 (1)	8.1	< 0.05	12	2.37 (0.81)	65
PDA-BiBB-PDEGMA^i^	4.46 (0.087)	17	1.27 (0.31)	17	0.39 (1)	17	< 0.05	50	–	–
**3a** in EtOH	–	–	–	–	–	–	–	–	0.47	

^a^Poly(diethylene glycol methyl ether methacrylate) (PDEGMA), 2-brom-2-methyl-propionylbromid (BiBB), polydopamine (PDA)

^b^Fluorescence lifetimes of the polymer

^c^Relative amplitude of the polymer decay component

^d^Contribution of the polymer decay component

^e^Fluorescence lifetime of **3a**

^f^Relative fluorescence intensities of **3a** and polymer

^g^Dry (in air) PDA-BiBB-PDEGMA brushes batch 1

^h^Dry (in air) PDA-BiBB-PDEGMA brushes batch 2 at 25 °C

^i^Wet (submersed in water) PDA-BiBB-PDEGMA brushes batch 2 at 25 °C

Similar to a detection of a submersed air–water interface by the lifetime of a reporter dye [58–60], an inverse problem to specify the properties of the reporter dye in the nanomaterial might be solved by a global analysis of the fluorescence decay. In the present work, a set of integral Eq. (2) for 60 fluorescence decays corresponding to the various compositions of PDA-BiBB-PDEGMA brushes in the presence and absence of aurone **3a** was fitted globally to specify the fluorescence decay time of the dye as well as those for the (auto)fluorescing polymer components. The results, obtained under the assumption that the values of *τ* of these components are independent of the composition of the brushes, are shown in Table 4.

The fluorescence kinetics of PDEGMA brushes itself (cf. SI, Fig. S9a and Table 4) decays triple-exponentially with the times *τ*_p_ = 5.97–6.08, 1.76–1.90 and 0.43–0.48 ns slightly depend on the sample batch covering 48–54% of polymer emission. The remaining fraction of intensive brush emission is short-lived, *τ*_*p*4_ < 0.05 ns, originating probably to a large extent from the PDA and BiBB layers underneath the brush. The fluorescence of PDA and PDA-BiBB films consists of only the short-lived component *τ*_*p*4_ (cf. SI, Fig. S9b and Table 4), implying that the three long-lived exponential terms *τ*_*p*1_, *τ*_*p*2_ and *τ*_*p*3_ (*τ*_*p*1–3_) are associated with the autofluorescence of PDEGMA. Furthermore, the contributions of these components to the total emission were in the same range for these PDEGMA samples (Table 4).

Wetting of the polymer with water led to substantial shortening of the decay times by ~ 35% from 6.08, 1.90 and 0.47 ns to 4.46, 1.27 and 0.39 ns keeping their contribution *A* and relative amplitudes *a* of the components almost unchanged (cf. SI, Fig. S8d (Table 4). This observation allowed us to conclude that the fluorescence of brushes originated from several emitters, which are quenched dynamically by water. The quenching efficiency *q* was estimated as a ratio of the corresponding decay times at wet (*τ*_*w*_) and dry (*τ*_*a*_) conditions (Eq. 3). For *τ*_*p*1–3_, the *q*-factors (1.4, 1.5, and 1.2) are close to each other supposing the same general fluorescence quenching mechanism, such as an internal conversion [61].

3 $$ q = \frac{{\tau_{a} }}{{\tau_{w} }} $$

The fluorescence of the wetted polymer brushes decayed faster than that of the dry polymer brushes, as shown in Fig. 8d. Because of the high dry/wet fluorescence lifetime contrast of ~ 35%, the autofluorescence of PDEGMA brushes might be used also to image the polymer (de)wetting with FLIM technique employing an averaged fluorescence lifetime as well as the one of the component.

The fluorescence decay of the polymer brushes loaded with aurone **3a** were fitted well with the five exponentials. Specifically, three long-lived components had the same decay times as *τ*_*p*1-3_ for the polymer itself, whereas a short-lived (< 0.05 ns) component and an additional fluorescence lifetime of 3.02 and 3.12 ns for the dried PDEGMA brushes and 2.37 ns for PDEGMA brushes submersed in water were observed (Table 4, Fig. 8a, b, c). This additional decay time was assigned to the fluorescence lifetimes of dye **3a** in dry and wet PDEGMA brushes. The *τ*_*d*_ component of the dye fluorescence was 71%, 139% and 80% of the long-lived *τ*_*p*1-3_ fluorescence of the brushes and 49%, 109% and 65% of the total PDEGMA brushes emission. Overall, the fluorescence of the dye was sufficiently strong to be detected and to estimate the fluorescence lifetime of the dye in dry as well as in wet polymer.

It has to be noted that the relative amplitude ratios *a*_2_*/a*_3_ (compare Table 4) increased by a factor of 3.2, 5.4, and 4.6 times in the presence of aurone **3a**. This effect is probably a result of more complex fluorescence kinetics of the dye in PDEGMA brushes when the dye has a fluorescence decay component close to *τ*_*p*2_. In that case, the part of long-lived fluorescence associated with the polymer contains also shorter-lived dye fluorescence. And the ratios of fluorescence intensities of dye to polymer of 71–139% and 49–109% should be considered as the lower-bound estimates.

The fluorescence of aurone **3a** in ethanol, as a polar protic solvent that mimics an aqueous medium, decays mono-exponentially with *τ*_d_ = 0.47 ns (Table 4 and Fig. 7b) and is much shorter (6.4 times) than in dry PDEGMA brushes. Because aurone **3a** does not fluoresce in water (Table 1), the decay time there cannot be estimated by direct measurements in this setup. It is expected that the value of *τ*_d_ in wetted PDEGMA will be between 3.02 and 0.47 ns or less than 0.47 ns. The experimental value of 2.37 ns supports this estimation and shows that the bound water in the polymer matrix possesses a lower *q*-factor than EtOH. This assertion is based on the observation that the quantum yield *Φ* of aurone **3a** in MeOH is lower than the one in less polar EtOH (Table 1). As the variation of *τ*_d_ by > 6 times for aurone **3a** when going from PDEGMA to aqueous media is higher than that of ATTO 655 (1.5–2 times), the aurone **3a** is suggested to be a feasible reporter dye for detection of the wetting of the polymer employing FLIM with somewhat higher contrast or a time-resolved fluorescence microscopy.

In aurone-loaded PDA-BiBB-PDEGMA the major fluorescing component (67%) is the polymer whereas the dye has only a minor contribution of 33% to the total emission. Hence, the detection of wetting of this polymer with this reporter dye in intensity images is less straightforward because the signal value for the wetted case does not exceed 49% of background fluorescence signal.

To solve the problem whether the dye signal stands out above the polymer background signal in time-resolved images, the convoluted with IRF exponential terms were plotted together with the overall decay kinetics (cf. SI, Fig. S10). Obviously, the short-lived partial polymer fluorescence components (*τ*_*p*3_ and *τ*_*p*4_) were higher than the component (*τ*_d_) for the dye at earlier time (*t* ≈ 0), moderate-lived component *τ*_*p*2_ at *t* < 1.12 ns and long-lived component *τ*_*p*1_ at *t* > 12.7 ns after the excitation maximum (*t* = 0) (cf. SI, Fig. S10a). A very similar behavior was observed in the fluorescence decay of aurone **3a** in another batch of brushes (cf. SI, Fig. S10b, c). With the dry brushes such time range is somewhat larger than for wet brushes. This observation indicates that somewhere in this time range the intensity of dye fluorescence can be even stronger than that of polymer. It will happen when there is a time range where the following condition as expressed in inequality (4) is satisfied.

4 $$ p\left( t \right)*\left( {a_{d} e^{{ - \frac{t}{{\tau_{d} }}}} - \mathop \sum \limits_{j = 1}^{4} a_{pj} e^{{ - \frac{t}{{\tau_{pj} }}}} } \right) > 0 $$

For aurone **3a** in dry PDA-BiBB-PDEGMA brushes a somewhat smaller time range from 2.2 to 12.4 ns is obeyed (Fig. 8b), at time > 0.66 ns (Fig. 8a) and in wet brushes at time > 1.68 ns (Fig. 8c). In this range the image contrast function *c*(*t*) was estimated, as the ratio of such signal excess (4) to the background intensity by Eq. (5). Figure 8a, b, c shows that *c*(*t*) is going over peak values of 0.55 at 7.0 ns, 2.9 at 10.7 ns and 2.5 at 8.0 ns, respectively. These results allow us to conclude that although the intensity image integrated over time possesses lower and even negative *c* = −0.51, 0.16 and −0.32 (Table 4), the image contrast factor in the time-resolved image reaches significant positive values, which are 3.1, 3.3 and 5.2 times higher than for intensity image. Therefore, time-resolved microscopy due to increasing the image contrast, which may be improved by factor of 3.1, offers an opportunity to image labelling dye above an emissive matrix background.

5 $$ c\left( t \right) = \frac{{p\left( t \right)*\left( {a_{d} e^{{ - \frac{t}{{\tau_{d} }}}} } \right)}}{{p\left( t \right)*\left( {\mathop \sum \nolimits_{j = 1}^{4} a_{pj} e^{{ - \frac{t}{{\tau_{pj} }}}} } \right)}} - 1 $$

## Conclusion

Three water-sensitive fluorescent amino-substituted aurone derivatives were synthesized. The dye **3a** possesses moderate fluorescence in aprotic solvents, low fluorescence in protic solvents and practically no fluorescence in aqueous solution. Because of its low water solubility, the dye **3a** is a feasible humidity probe for polymers, by non-covalent embedment, as shown in this work, or potentially also by covalent attachment to the polymer. The stable PDEGMA polymer brushes with thickness about 200 nm was shown to be grafted from a Ti surface by SI-ARGET ATRP surface polymerization of DEGMA in Ar-saturated solution. The brushes loaded with aurone **3a** show rather strong fluorescence of the dye comparable with the autofluorescence of the PDEGMA brushes, the subjacent thin BiBB initiation, and the anchor PDA layers. With the global analysis of a set of the fluorescence decays, the lifetimes of the complex multicomponent materials can be deconvoluted. It has been shown that dry PDA-BiBB-PDEGMA brushes loaded with aurone **3a** possess five-exponential fluorescence decay with the characteristic decay times ~ 3.0 ns for the dye, and ~ 6.0, 1.8 and 0.45 ns for PDEGMA brushes and < 0.05 ns for subjacent PDA and BiBB layers. Because of the strong decrease of fluorescence lifetime down to ~ 2.4 ns for dye and to 4.5, 1.3 and 0.40 ns for matrix submersed in water, both dye and matrix fluorescence are a feasible reporter fluorophores for the study of wetting processes of the polymer employing the fluorescence lifetime imaging. The contrast of the intensity images of aurone **3a** being rather low because of background PDA-BiBB-PDEGMA matrix fluorescence increases by a factor of 3.1 in the time-resolved imaging at certain time delay after excitation (7 ns). The incorporation of fluorescent dye **3a** in PDA-BiBB-PDEGMA brushes seems to be a promising versatile system for an investigation of brushes (de)wetting dynamics employing fluorescence lifetime imaging as well as nanosecond time-resolved fluorescence imaging microscopy.

## Supplementary Information

Below is the link to the electronic supplementary material.

Supplementary file1 (PDF 1911 KB)

## Data Availability

The data supporting this article have been included as part of the Supplementary Information and will be made available on the open access depository Zenodo (DOI: 10.5281/zenodo.20509532).
