# Supplementary material for: Unveiling the fluorescence lifetime changes of a solvatochromic aurone probe dye upon wetting in an emissive polymer brush matrix by deconvolution of TCSPC data
Source: Eur Phys J E Soft Matter. 2026 Jul 16;49(8):60. doi: 10.1140/epje/s10189-026-00597-5 (PMC13375835; doi:10.1140/epje/s10189-026-00597-5)
Supplement: Supplementary file 1 — Supplementary file1 (PDF 1911 KB) [file 10189_2026_597_MOESM1_ESM.pdf]

Supplementary Material

Unveiling the fluorescence lifetime changes of a solvatochromic aurone probe dye upon wetting in an emissive polymer brush matrix by deconvolution of TCSPC data

Jonah L. Decker,[1] Sören Steup,[2] Sergey I. Druzhinin,[1]* Heiko Ihmels,[2]* Holger Schönherr[1]*

[1] *Physical Chemistry I, Department of Chemistry and Biology, and Research Center of Micro and Nanochemistry and (Bio)Technology (Cμ), University of Siegen, Adolf-Reichwein-Str. 2, 57076 Siegen, Germany*

[2] *Organic Chemistry II, Department of Chemistry and Biology, and Research Center of Micro and Nanochemistry and (Bio)Technology (Cμ), University of Siegen, Adolf-Reichwein-Str. 2, 57076 Siegen, Germany*

1. **Synthesis of aurone derivative 3b**

(*Z*)-3-Oxo-2-((2,3,6,7-tetrahydro-1*H*,5*H*-pyrido [3,2,1-*ij*]quinolin-9-yl)methylene)-2,3dihydrobenzofuran-6-yl methacrylate **3b**

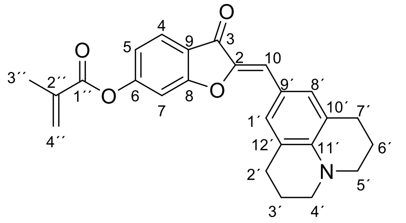

To a solution **3a** (100 mg, 0.30 mmol) and Et_3N (46 mg, 35 μL, 0.45 mmol) in CH_2Cl_2 (1 mL) was added methacryloyl chlorid (38 mg, 35 μL, 0.36 mmol) at 0 °C under argon atmosphere, and the reaction mixture was stirred for 45 min at 0 °C. The mixture was poured into water (20 mL) and extracted with CH_2Cl_2 (2 × 20 mL). The solvent was removed in vacuum. The product **3b** was obtained by crystallization from MeOH as dark red crystals (73 mg, 0.18 mmol, 61%); mp < 210 °C (dec.). ^{1}H NMR (500 MHz, $CDCl_3$): δ = 2.02–1.93 (m, 4H, 3´-H, 6´-H), 2.08–2.09 (m, 3H, CH_3), 2.78 (t, ^3J = 6.4 Hz, 4H, 2´-H, 7´-H), 3.32–3.25 (m, 4H, 5´-H, 6´-H), 5.84–5.79 (m, 1H, 3´´-H), 6.38–6.39 (m, 1H, 3´´-H), 6.84 (s, 1H, 10-H), 6.94 (dd, ^3J = 8.3 Hz, ^4J = 1.9 Hz, 1H,

5-H), 7.21 (d, ^4J = 1.8 Hz, 1H, 7-H), 7.38 (s, 2H, 1′-H, 8′-H), 7.81 (d, ^3J = 8.3 Hz, 1H, 4-H). – ^{13}C NMR (125 MHz, $CDCl_3$): δ = 18.5 (CH_3), 21.6 (C3′, C6′), 27.9 (C2′, C7′), 50.2 (C4′, C5′), 106.7 (C7), 116.6 (C10), 116.9 (C5), 118.9 (9′), 120.5 (C9), 121.3 (C10′, C12′), 125.1 (C4), 128.2 (C3′′), 131.6 (C1′, C8′), 135.7 (C2′′), 145.1 (C11′), 145.3 (C2), 156.8 (C6), 165.2 (C8), 165.6 (C1′′), 182.5 (C3). – MS (ESI$^+$): *m/z* (%) = 402 (40) [M^+ + H], 334 (100) [$M^+ - C_4H_6O$]. – El. Anal. for $C_{25}H_{23}NO_4$, calcd. (%), C 74.80, H 5.77, N 3.49, found (%) C 74.75, H 5.96, N 3.18.

Synthesis of aurone derivative **3c**

(*Z*)-2-(2-(2-((3-oxo-2-((2,3,6,7-Tetra–hydro-1*H*,5*H*-pyrido[3,2,1-*ij*]quinolin-9-yl)methylene)-2,3-dihydrobenzofuran-6-yl)oxy)ethoxy)ethoxy)ethyl methacrylate **3c**

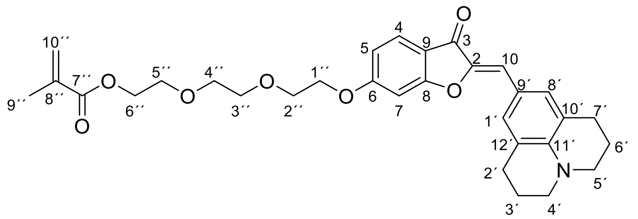

To a mixture of **3a** (274 mg, 1.62 mmol), K_2CO_3 (134 mg, 0.81 mmol) and KI (50 mg, 0.81 mmol) in anhydrous DMF (5 mL) was added 2-(2-(2-chloroethoxy)ethoxy)ethyl methacrylate (224 mg, 1.62 mmol) , and the reaction mixture was stirred for 16 h at 90 °C. After cooling the mixture to r.t., CH_2Cl_2 (10 mL) was added, and the solution was washed with water (10 mL) and brine (10 mL). The product was purified by column chromatography (SiO_2, hexane/EtOAc: 1/1, R_f = 0.44) and obtained as black oil (15 mg, 0.8 mmol, 10%). ^{1}H NMR (600 MHz, DMSO-d_6): δ = 1.92–1.85 (m, 8H), 2.70–2.73 (m, 4H, 2′-H, 7′-H), 3.25–3.27 (m, 2H), 3.64–3.55 (m, 4H, 5′-H, 6′-H), 3.69–3.65 (m, 2H), 3.82–3.77 (m, 2H), 4.23–4.18 (m, 2H), 4.28–4.24 (m, 2H), 5.65–5.66 (m, 1H, 9′′-H), 6.01–6.02 (m, 1H, 9′′-H), 6.62 (s, 1H, 10-H), 6.80 (dd, ^3J = 8.5 Hz, ^4J = 2.1 Hz, 1H, 5-H), 7.09 (d, ^4J = 2.1 Hz, 1H, 7-H), 7.36 (s, 2H, 1′-H, 8′-H),

7.60 (d, ^3J = 8.5 Hz, 1H, 4-H). – ^{13}C NMR (150 MHz, DMSO-d_6): δ = 18.0 (CH_3), 21.0 (C3′, C6′), 21.7 (-CH_2-O), 24.1 (-CH_2-O), 27.2 (C2′, C7′), 49.3 (C4′, C5′), 63.7 (-CH_2-O), 68.6 (-CH_2-O), , 78.7 (-CH_2-O), 97.2 (C7), 112.6 (C5), 113.8 (C10), 114.9 (C9), 117.8 (C9′), 120.7 (C10′, C12′), 124.7 (C9′′), 125.7 (C4), 130.7 (C1′, C8′), 135.8 (C2), 144.4 (C11′), 165.5 (C8), 166.6 (C6), 180.4 (C3). – MS (ESI$^+$): *m/z* (%) = 532 (60) [M^+ – H], 531 (100) [M^+ – 2 H]. – El. Anal. for $C_{31}H_{35}NO_7$, calcd. (%), C 69.78, H 6.61, N 2.62, found (%) C 69.99, H 6.67, N 2.42.

2. Absorption and emission spectra

Solutions were prepared for each measurement from stock solutions of **3a** in MeOH (c = 1.0 mM). Aliquots of the stock solution were thoroughly evaporated under a stream of nitrogen, and the residue was redissolved in the respective solvent or solvent mixture. The absorption spectra were recorded in a range of 200–650 nm with a collection rate of 300 nm/min.

Determination of fluorescence quantum yields

Fluorescence quantum yields (Φ) were determined at 25°C in dilute solutions with an absorbance below 0.1 at the excitation wavelength. Coumarin 153 (λ_{ex} = 412 nm) in EtOH was used as a standard (Φ = 0.38) [1]. The excitation slit was adjusted to 2.5 nm and the emission slit was adjusted to 5 nm, the detector voltage was set to 600 V, and the collection rate was adjusted to 120 nm/min.

The excitation wavelengths were set to 421 nm with a detection range from 430–750 nm. The relative fluorescence quantum yields, Φ, were determined according to Eq. (S1).

$$\Phi_X = \frac{F_X A_S}{F_S A_X} \cdot \frac{n_X^2}{n_S^2} \cdot \Phi_S \qquad \text{(S1)}$$

F is the integral of the spectral response corrected emission band, A is the absorbance at the excitation wavelength and n is the refraction index of the solution, the indices X and S indicate the analyte (X) and standard (S) solution. The estimated error is ±10% of the given values.

3. NMR Spectra

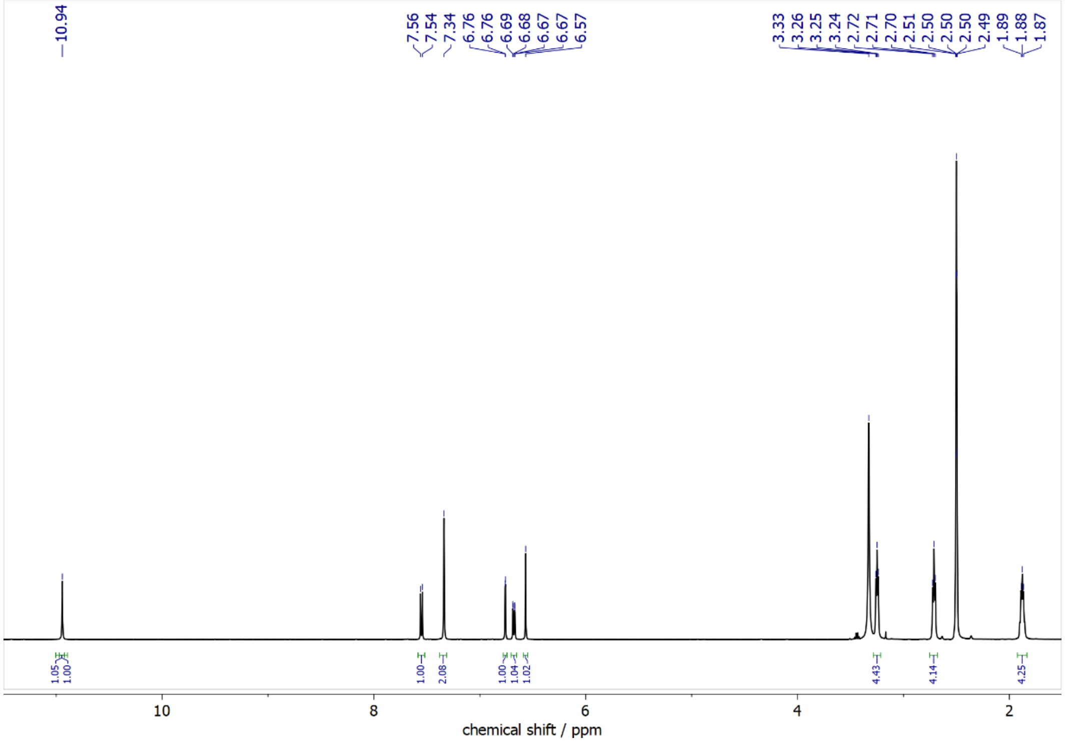

Fig. S1. ^{1}H NMR (600 MHz) spectrum of **3a** in DMSO-d_6.

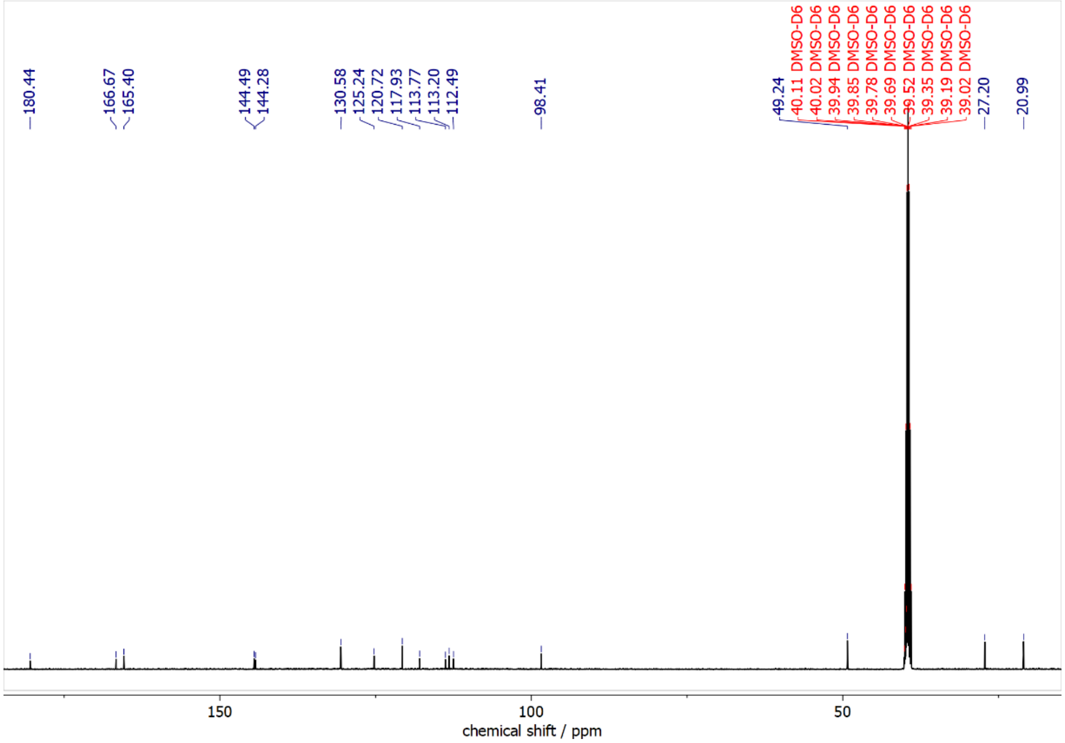

Fig. S2. ^{13}C NMR (600 MHz) spectrum of **3a** in DMSO-d_6.

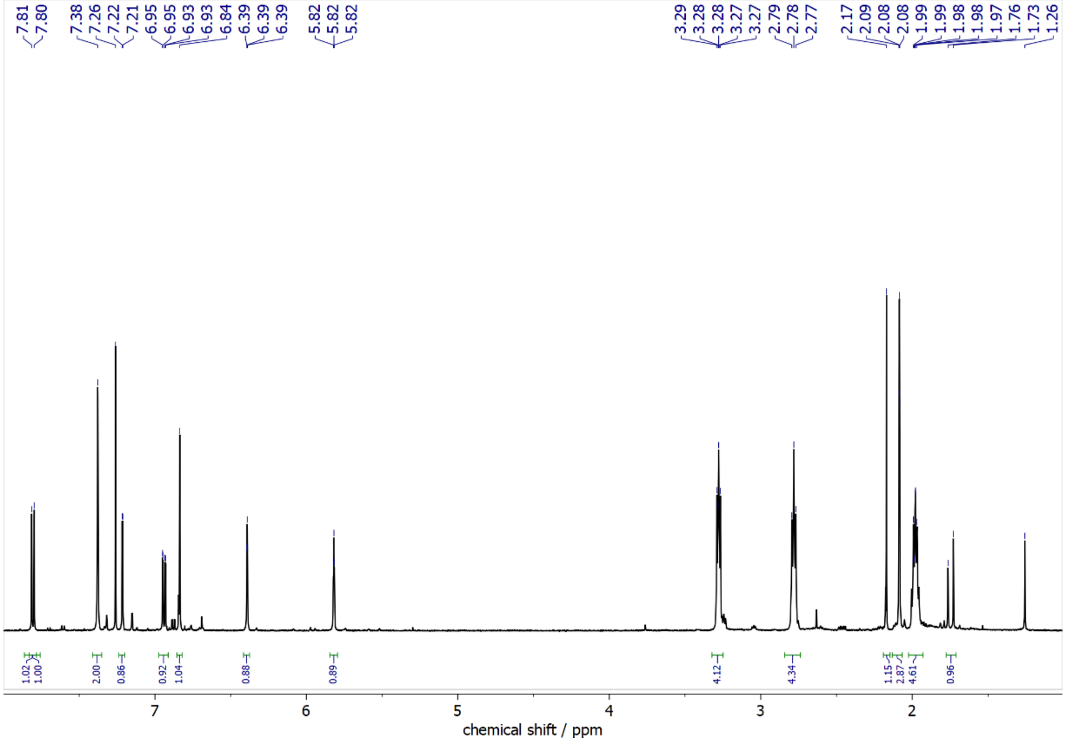

Fig. S3. ^{1}H NMR (500 MHz) spectrum of **3b** in $CDCl_3$.

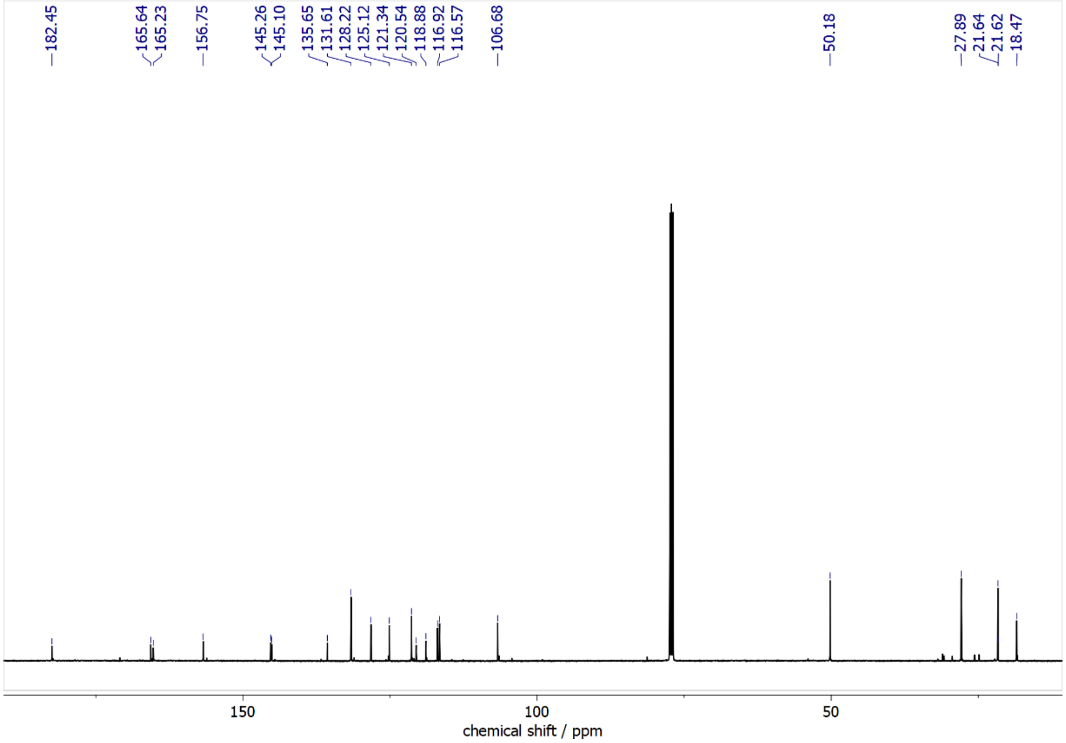

Fig. S4. ^{13}C (500 MHz) spectrum of **3b** in $CDCl_3$.

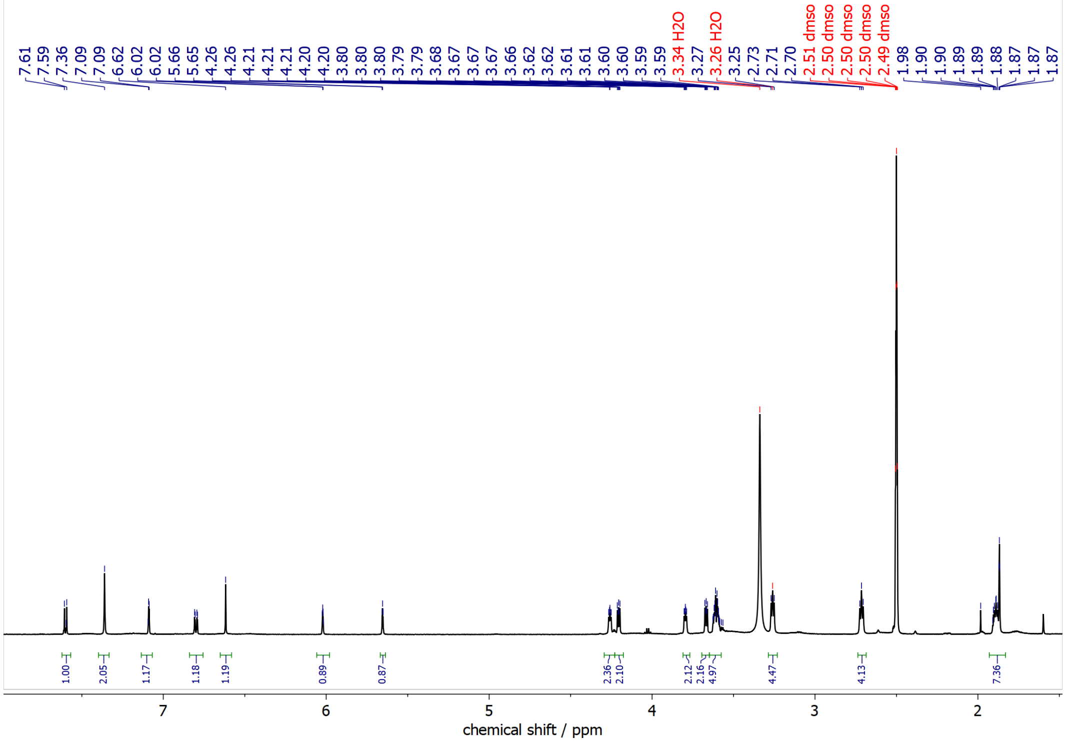

Fig. S5. ^{1}H NMR (600 MHz) spectrum of **3c** in DMSO-d_6.

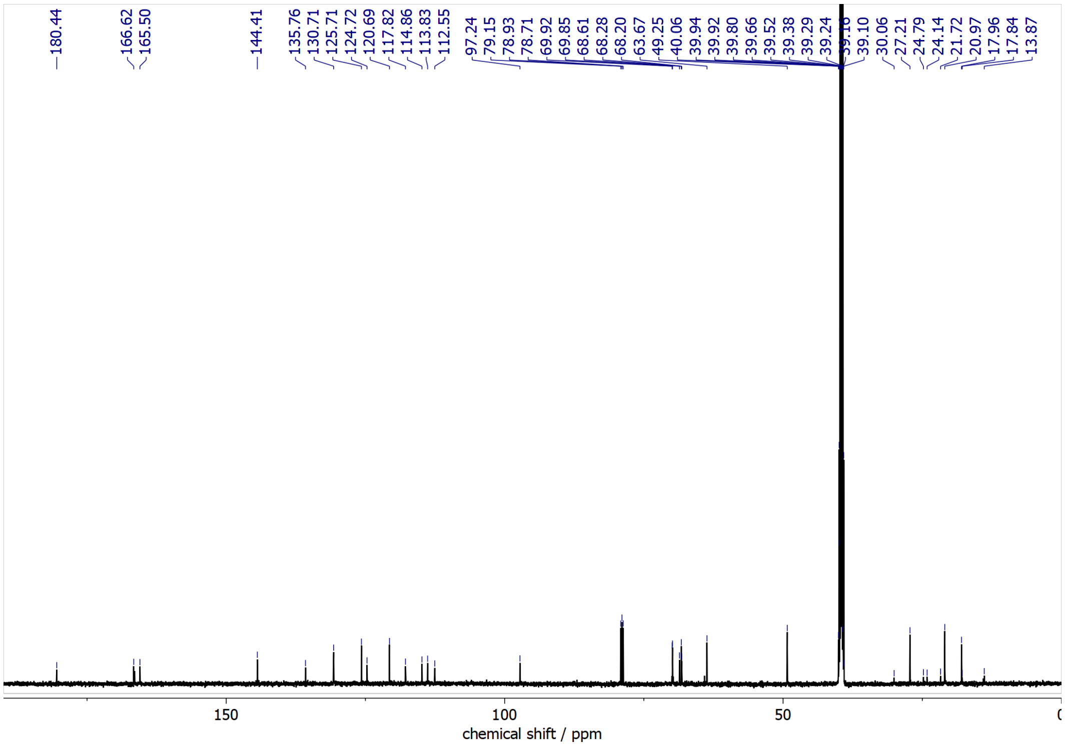

Fig. S6. ^{13}C NMR (600 MHz) spectrum of **3c** (600 MHz) in DMSO-d_6.

4. Synthesis of PDEGMA brushes

Materials

2-Propanol (>99.9%, CHEMSOLUTE, Germany), titanium granules (99.8 %, Chempur, Germany), ethanol (denatured, >99.2%, CHEMSOLUTE, Germany) dopamine hydrochloride (98%, Sigma-Aldrich, Germany), tris-HCl buffer (1M, Jena Bioscience, Germany), triethylamine (TEA, ≥99.5%, Sigma-Aldrich, Germany), α-bromoisobutyryl bromide (BiBB, >98.0%, TCI, Japan), dichloromethane (≥99.8%, Thermo Fisher, Germany), ethyl acetate(≥99.8%, Thermo Fisher, Germany), methanol (≥99.8%,Thermo Fisher, Germany), 2,2'-bipyridyl (≥99%, Sigma-Aldrich, Germany), copper(II) chloride dihydrate (AppliChem, Germany), L-ascorbic acid (99%, Sigma-Aldrich, Germany), ethanol (absolute, VWR, Germany) were used as received.

The Milli-Q water with a resistivity of 18.2 MΩ cm was taken from a Millipore Direct Q8 system (Millipore, Schwalbach, Germany) containing a Millimark Express 40 filter (Merck, Germany).

Di(ethylene glycol) methyl ether methacrylate (DEGMA, 95%, Sigma-Aldrich, Germany) was purified in a column filled with neutral aluminum oxide (activity stage I, Merck, Germany) and basic aluminum oxide (activated, Brockmann I, Sigma-Aldrich, Germany).

Substrate preparation

The titanium substrates were prepared by cutting glass substrates into 1.5 cm x 2.5 cm pieces. Those glass substrates were cleaned in a sonication bath once in soap water for 10 min and once in Milli-Q water. Subsequently, the samples were washed in boiling 2-propanol. After drying, the substrates were coated by electron beam evaporation under high vacuum (Edwards E306 coating system, Moorfield, UK) with 100 nm of titanium. For the microscopy measurements cover glasses (Menzel Gläser, Fisher Scientific, Waltham, USA) of 20 mm x 20 mm size were used. These substrates were cleaned and coated as descripted previously, but coated with only 5 nm of titanium to maintain transparency of the samples.

Polydopamine deposition

The titanium samples were rinsed extensively with ethanol and Milli-Q water and dried in a stream of nitrogen. After further cleaning of the substrates in the UV/Ozone cleaner (Bioforce Nanoscience, Ames, USA) for 20 min, they were immersed in an aqueous dopamine solution (2 mg per mL of dopamine hydrochloride in 10 mM tris-HCl buffer, pH 8.5) and kept on a shaker for 1h. Afterwards, the samples were cleaned in Milli-Q water by sonicating for 10 min and drying in the nitrogen stream.

BiBB attachment

The samples functionalized with polydopamine were immersed in 30 mL of anhydrous CH_2Cl_2 and cooled in an ice bath. TEA (500 µL) and diluted BiBB (250 µL in 3 mL anhydrous CH_2Cl_2) were successively added dropwise to the solution. The reaction was performed in an argon atmosphere for 1 h while the flask was sealed by paraffin film. Afterwards, the substrates were cleaned with CH_2Cl_2, ethyl acetate and water by sonicating for 3 min and drying in the nitrogen stream.

Polymerization of PDEGMA brushes

To synthesize the PDEGMA brushes, SI-ARGET ATRP was used. A mixture of Milli-Q water (19.2 mL), methanol (20 mL) and 2,2'-bipyridine (50 mg) was stirred for 15 min under constant argon flow. Afterwards, DEGMA (3.5 mL) and a aqueous $CuCl_2$ solution (0.4 mL, 0.04 M) were added. After stirring the solution for 10 min under argon flow, a aqueous solution of L-ascorbic acid (0.4 mL, 0.4 M) was added. After 1 min of stirring, the reaction solution was transferred into the reaction chamber, containing the substrates functionalized with the initiation layer in an argon atmosphere to perform 30 min of polymerization.

To stop the reaction, the samples were transferred into a beaker containing a mixture of methanol and Milli-Q water (ratio 1:1). Finally the samples were rinsed with Milli-Q water and dried in a nitrogen stream.

5. Ellipsometry

To analyze the dry thickness of the polymer brushes, an alpha-SE ellipsometer (J.A. Woollam Co., Inc., Lincoln, USA) was used. Three different incidence angles: 65°, 70°, and 75° of the light with wavelengths between 380 nm and 900 nm were used. For each thickness determined by ellipsometry, the arithmetic mean and standard deviation of nine measurements from three

different samples were calculated. The data processing was done in Complete EASE (version 6.66). The thicknesses of polydopamine and PDA-BiBB layers were fitted with a Cauchy model on top of the Ti substrate. The thickness and Cauchy coefficients A, B and C were fitted. The polymer brush samples were fitted with a Sellmeier model which included the Ti background, one layer including a Cauchy model, with a fixed thickness measured beforehand for the PDA-BiBB layer of the sample, while Cauchy coefficients A, B and C were fitted and a second layer fitted with the Sellmeier model, where the thickness, index offset, amplitude, center energy and the position of a pole in the infrared were fitted to determine the thickness of the polymer brushes.

6. Contact angle measurements

The static and dynamic water contact angle measurements were determined at 20 °C using an OCA-15 contact angle microscope (Dataphysics, Fildersatadt, Germany) with a 500 µL syringe (Hamilton, Bonaduz, Swiss) and the SCA20 software (version 5.0.22). A volume of 2 µL was used for the droplet of Milli-Q water to measure the static contact angle by sessile drop method. To determine the dynamic contact angles, additional 18 µL of water were pumped into the 2 µL droplet with a rate of 2 µL/s and after a delay time of 5 s the 18 µL were sucked out of the droplet with a rate of 2 µL/s. This process of pumping 18 µL into the droplet and sucking it out was repeated once. During pumping in the water, the highest contact angle of the first cycle was determined as advancing contact angle. However outliers due to initial pinning of the three-phase contact line were neglected. The lowest contact angle of the first cycle was determined as receding contact angle. For each contact angle the arithmetic mean and the standard deviation of

nine measurements from three samples were measured and on each sample three measurements were performed

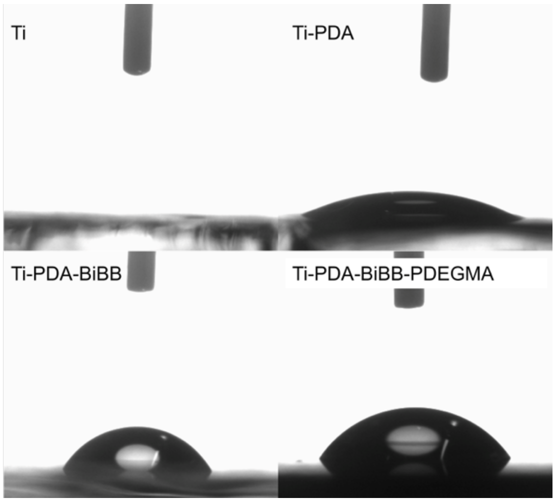

Fig. S7. Water contact angle measurements by sessile drop method on titanium (Ti), on polydopamine (Ti-PDA), on polydopamine functionalized with BiBB (Ti-PDA-BiBB) and on PDEGMA polymerized from PDA-BiBB (Ti-PDA-BiBB-PDEGMA).

7. Grazing angle Fourier transform infrared spectroscopy

The Fourier transform infrared spectroscopy (FTIR) measurements were conducted using a Vertex Neo R spectrometer (Bruker Optics, Ettlingen, Germany) together with a VeeMAX-II Variable Specular Reflectance Accessory (PIKE Technologies, Fitchburg, USA). The grazing angle measurements were performed at an incidence angle of 73° with p-polarized light at room temperature under vacuum. 1024 scans were acquired with the aperture set to 8 mm and the spectral range from 600 cm^{-1} to 4000 cm^{-1}. A titanium sample cleaned as described earlier for the polydopamine deposition, was measured as background and a baseline correction was performed using the concave rubber band method by using the OPUS 9.0 SP1 software for analysis.

8. XPS Spectra

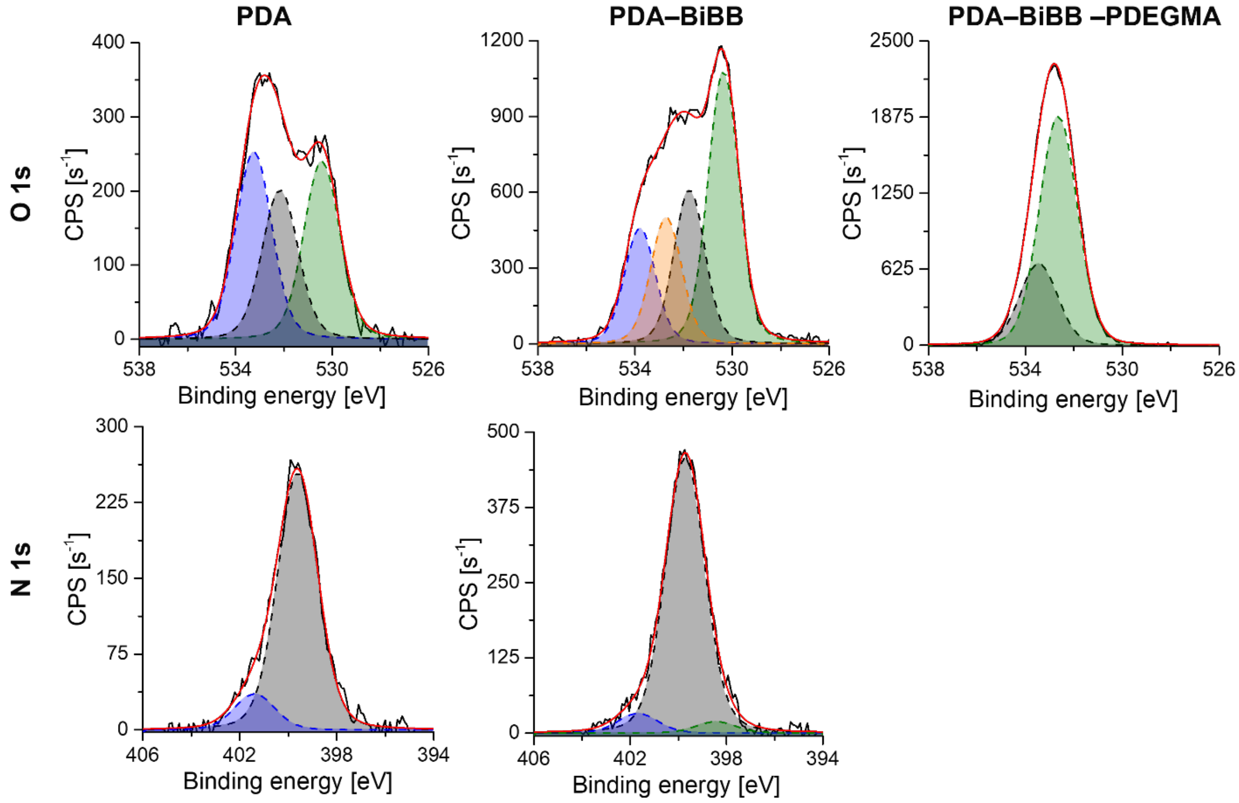

Fig. S8. X-ray photoelectron high resolution spectra of O 1s and N 1s from polydopamine (PDA), PDA functionalized with BiBB (PDA-BiBB) and PDEGMA polymerized from PDA-BiBB (PDA-BiBB-PDEGMA).

9. TCSPC data

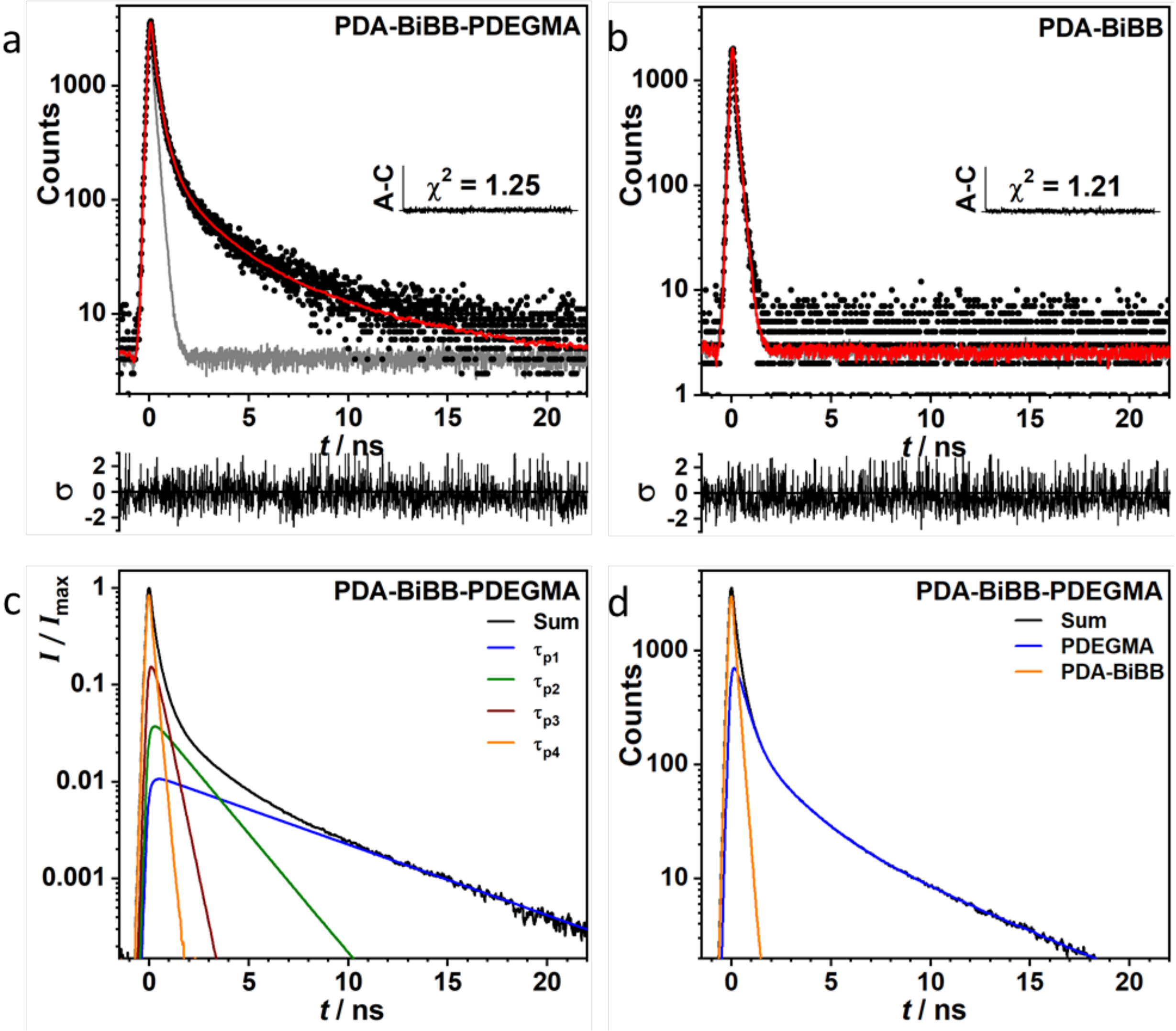

Fig. S9 (a, b) TCSPC fluorescence decay of PDA-BiBB-PDEGMA brushes batch 1 (a) and subjacent PDA-BiBB layer (b) at 22 °C at the excitation 485 nm. TCSPC panel: fluorescence decay, black dots, instrument response function (IRF), gray solid line, and fit, red solid line, (top panel) with the corresponding residuals (bottom panel) and autocorrelation function plot (inset in the top panel) are shown. In the panel, the χ^2 values are provided. (c) The components τ_{p1}, τ_{p2}, τ_{p3} and $\tau_{p4}$of fluorescence kinetics (a) as well as the sum of them. (d) The slow (sum of τ_{p1}, τ_{p2} and τ_{p3} components) fluorescence decay of PDA-BiBB-PDEGMA brushes, fast (τ_{p4} component) decay of PDA-BiBB subjacent layers and the sum of them.

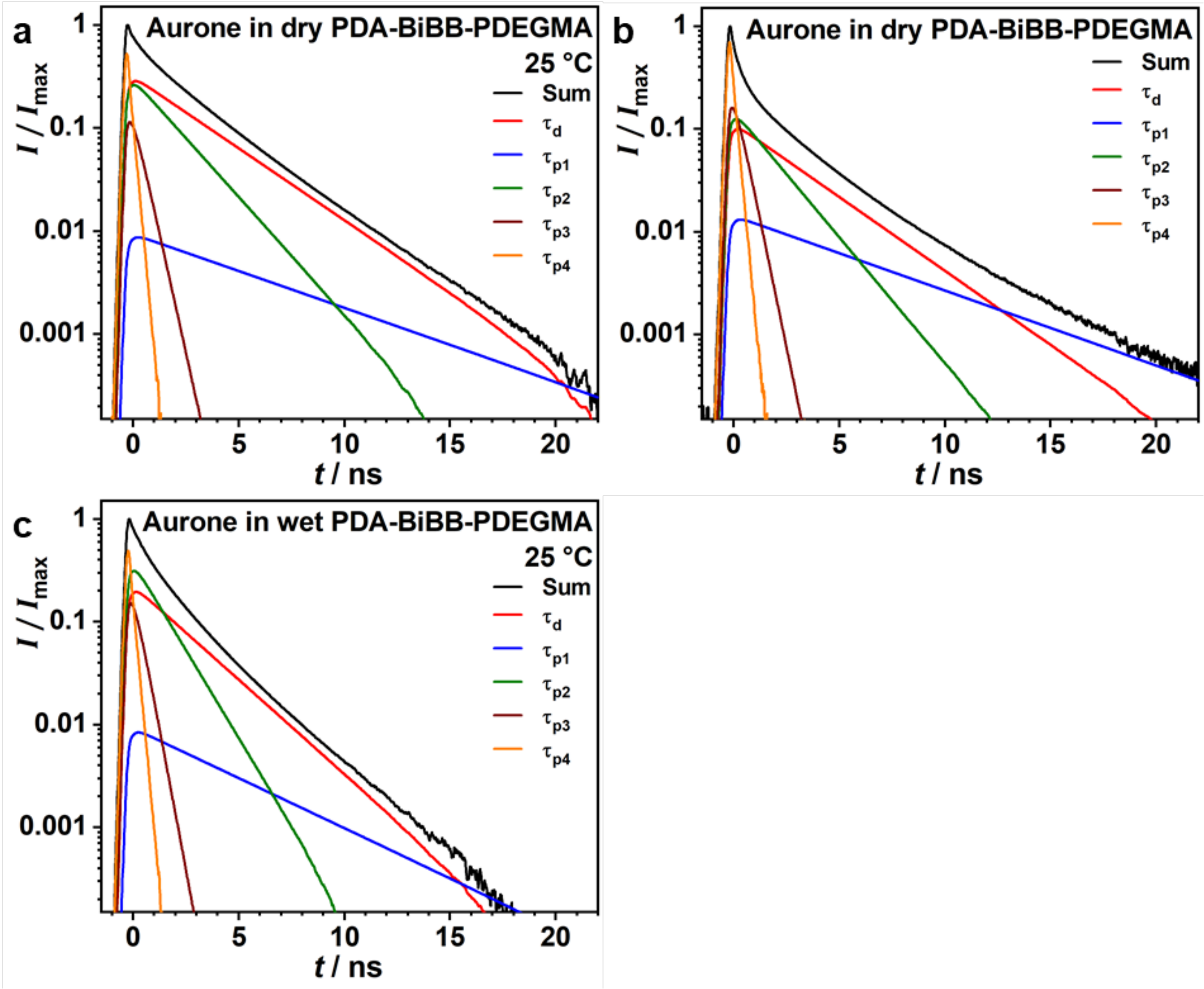

Fig. S10 Deconvolution of fluorescence decay of PDA-BiBB-PDEGMA brushes loaded with aurone **3a** at the excitation 485 nm. The components of fluorescence kinetics: aurone **3a** (τ_d), polymer brushes (τ_{p1}, τ_{p2}, τ_{p3} and τ_{p4}) and the sum of them. (a) Brushes batch 2 in air (dry) at 25 °C. (b) Brushes batch 1 in air (dry) at 22 °C. (c) Brushes batch 2 submersed in water (wet) at 25 °C.

10. Analysis of solvatochromic properties of 3a

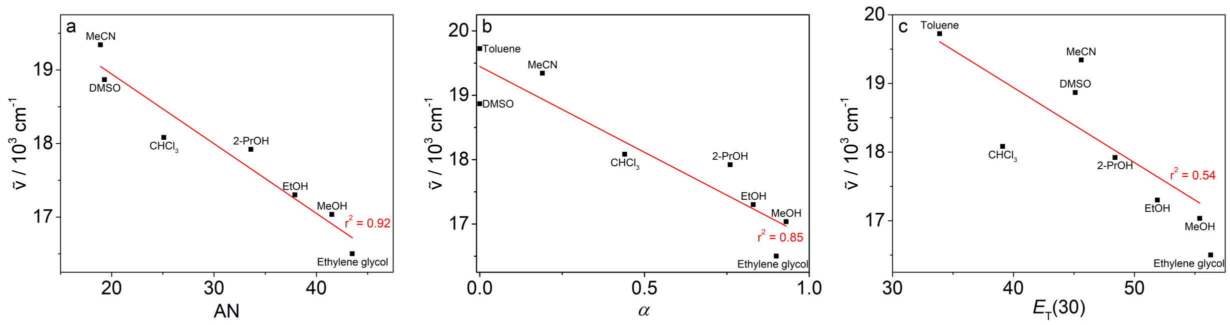

Fig. S11 Plot of the fluorescence maximum (in cm^{-1}) of **3a** in different solvents *versus* the solvent parameters AN [2] (a), α [2] (b), and $E_T(30)$ [2](c). The red lines indicate the result of linear regression analysis with the corresponding coefficient of dermination r^2.

11. References

[1] A.M. Brouwer, Standards for photoluminescence quantum yield measurements in solution (IUPAC Technical Report). Pure Appl. Chem. **83**, 2213 (2011). http://dx.doi.org/10.1351/PAC-REP-10-09-31

[2] C. Reichardt, in *Solvents and Solvent Effects on Organic Chemistry*, Wiley-VCH, Weinheim, 3rd edn., 2003; and references cited therein.
